# Functional Analysis of the GPI Transamidase Complex by Screening for Amino Acid Mutations in Each Subunit

**DOI:** 10.3390/molecules26185462

**Published:** 2021-09-08

**Authors:** Si-Si Liu, Fei Jin, Yi-Shi Liu, Yoshiko Murakami, Yukihiko Sugita, Takayuki Kato, Xiao-Dong Gao, Taroh Kinoshita, Motoyuki Hattori, Morihisa Fujita

**Affiliations:** 1 Key Laboratory of Carbohydrate Chemistry and Biotechnology, Ministry of Education, School of Biotechnology, Jiangnan University, Wuxi 214122, China; lss6442021@163.com (S.-S.L.); liuyishi@jiangnan.edu.cn (Y.-S.L.); xdgao@jiangnan.edu.cn (X.-D.G.); 2 State Key Laboratory of Genetic Engineering, Collaborative Innovation Center of Genetics and Development, Shanghai Key Laboratory of Bioactive Small Molecules, Department of Physiology and Neurobiology, School of Life Sciences, Fudan University, Shanghai 200438, China; jinf16@fudan.edu.cn (F.J.); hattorim@fudan.edu.cn (M.H.); 3 Research Institute for Microbial Diseases, Osaka University, Suita 565-0871, Osaka, Japan; yoshiko@biken.osaka-u.ac.jp (Y.M.); tkinoshi@biken.osaka-u.ac.jp (T.K.); 4 WPI Immunology Frontier Research Center, Osaka University, Suita 565-0871, Osaka, Japan; 5 Institute for Protein Research, Osaka University, Suita 565-0871, Osaka, Japan; sugita.yukihiko.8w@kyoto-u.ac.jp (Y.S.); tkato@protein.osaka-u.ac.jp (T.K.)

**Keywords:** glyco-diosgenin, GPI-anchored proteins, GPI-transamidase, protein purification, single particle

## Abstract

Glycosylphosphatidylinositol (GPI) anchor modification is a posttranslational modification of proteins that has been conserved in eukaryotes. The biosynthesis and transfer of GPI to proteins are carried out in the endoplasmic reticulum. Attachment of GPI to proteins is mediated by the GPI-transamidase (GPI-TA) complex, which recognizes and cleaves the C-terminal GPI attachment signal of precursor proteins. Then, GPI is transferred to the newly exposed C-terminus of the proteins. GPI-TA consists of five subunits: PIGK, GPAA1, PIGT, PIGS, and PIGU, and the absence of any subunit leads to the loss of activity. Here, we analyzed functionally important residues of the five subunits of GPI-TA by comparing conserved sequences among homologous proteins. In addition, we optimized the purification method for analyzing the structure of GPI-TA. Using purified GPI-TA, preliminary single particle images were obtained. Our results provide guidance for the structural and functional analysis of GPI-TA.

## 1. Introduction

Protein anchoring by glycosylphosphatidylinositol (GPI) is a conserved posttranslational modification in eukaryotes [1,2,3,4]. More than 150 proteins such as adhesion molecules, receptors, ligands, hydrolytic enzymes, and prion proteins are in the form of GPI-anchored proteins (GPI-APs) on the cell surface in mammalian cells. GPI confers unique properties to proteins. For example, GPI-APs are associated with lipid rafts and dynamic membrane domains composed of sphingolipids and cholesterol [5]. The association with lipid rafts regulates polarized transport, signal transduction, and endocytosis [6,7,8,9].

**Figure 1:**
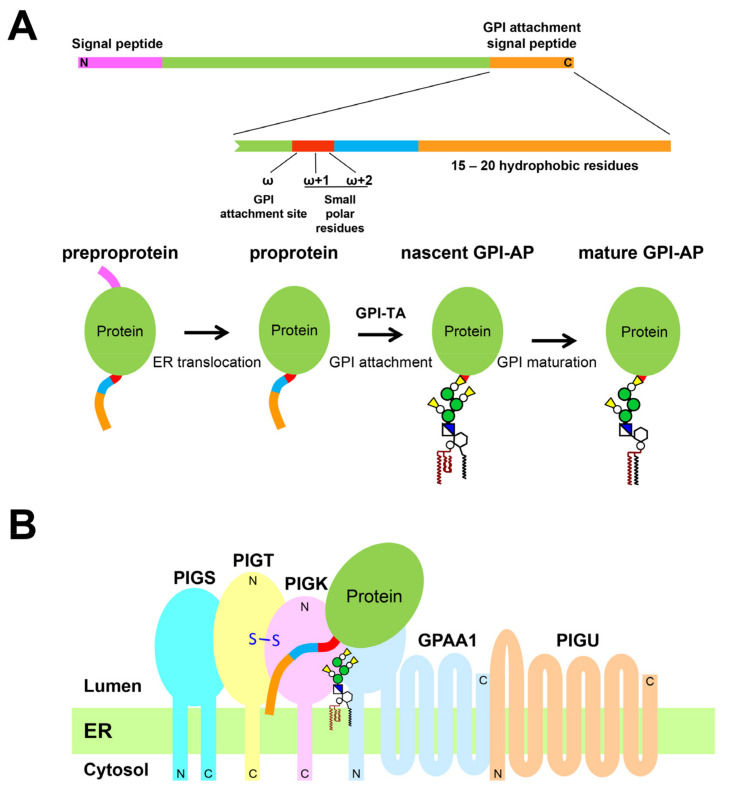
GPI attachment to proteins mediated by the GPI-TA complex. (**A**) Schematic representation of a GPI precursor protein that possesses an N-terminal signal peptide for ER translocation (pink) and a C-terminal GPI attachment signal (orange). The GPI-attachment signal consists of (1) ω, ω + 1, and ω + 2 amino acid residues with short side chains (red); (2) five to ten hydrophilic amino acids (blue); and (3) 15–20 hydrophobic amino acids at the C-terminus (orange). When a GPI precursor protein (preproprotein) is translocated into the ER, the N-terminal signal peptide is cleaved by signal peptidases. By reaction of the GPI-TA complex, the C-terminal GPI attachment signal is cleaved from proprotein at the ω-site, which is ligated to the preassembled GPI via an amide bond, generating a nascent GPI-AP. The GPI moiety is remodeled during transport, and mature GPI-AP is generated. (**B**) Schematic representation of the GPI-TA, which is a complex with five subunits localized on the ER membrane. PIGK is a catalytic subunit and forms an intermolecular disulfide bond with PIGT. GPAA1 may catalyze the formation of the amide bond between the ω-site and a bridging ethanolamine-phosphate (EtNP) on GPI. PIGU may be related to recognizing the GPI attachment signal or the lipid portion of GPI. The function of PIGS is still not clear.

GPI biosynthesis and transfer to proteins are carried out on the endoplasmic reticulum (ER) membrane. The synthesis of GPI is started from phosphatidylinositol (PI) by the stepwise addition of sugars, a fatty acid, and ethanolamine-phosphates (EtNPs). GPI precursor proteins, which have an N-terminal signal sequence for ER translocation and a GPI attachment signal peptide at the C-terminus, are separately synthesized (Figure 1A). Once the proteins are translocated into the luminal side of the ER, the N-terminal signal sequence is cleaved by signal peptidases, and the C-terminal GPI attachment signal peptide is recognized by GPI-transamidase (GPI-TA), which transfers a GPI to the proteins [10].

Mammalian GPI-TA is a multi-subunit complex consisting of five subunits: PIGK, GPAA1, PIGT, PIGS, and PIGU (Figure 1B). PIGK is the catalytic subunit of GPI-TA, and is homologous with members of the cysteine protease family with transaminase activity [11]. PIGK likely cleaves the GPI attachment signal between ω and ω + 1 sites and forms the thioester bond on the catalytic Cys residue to generate an enzyme–substrate intermediate. GPI is then transferred to the newly exposed C-terminus of the protein. This reaction may be mediated by GPAA1, which is structurally similar to M28-type aminopeptidase [3,12]. GPAA1 is proposed to catalyze the formation of an amide bond between the ω-site and the bridging EtNP on the GPI complete precursor [13]. Mammalian PIGK and PIGT form an intermolecular disulfide bond that stabilizes GPI-TA formation. The loss of this disulfide bond reduces the activity of GPI-TA [14]. Through sequence homology analysis, PIGT is predicted to form an open-ring β-propeller structure with an additional α-helical N-terminal hook that can embrace another subunit [15,16]. PIGU is homologous with other GPI biosynthetic enzymes (such as PIGW and PIGM), suggesting that it recognizes the lipid portion of GPI [17]. Lacking PIGU, other subunits (PIGK, GPAA1, PIGT and PIGS) still form a complex, but have no activity. PIGS is an indispensable subunit of GPI-TA activity, although its role is unclear.

Mutations in the GPI-TA genes *PIGK* [18], *GPAA1* [19], *PIGT* [20], *PIGS* [21], and *PIGU* [22] have been reported to cause inherited GPI deficiencies (IGDs), which are characterized by intellectual disability, seizures, hypotonia, and facial deformities [23]. In addition, somatic *PIGT* mutations in hematopoietic stem cells cause a type of paroxysmal nocturnal hemoglobinuria (PNH) that has autoinflammatory features such as aseptic meningitis [24,25]. On the other hand, dysregulated overexpression of GPI-TA subunits is known to promote various cancers including bladder cancer, lymphoma, and breast cancer [26]. Therefore, understanding the molecular and structural bases of GPI-TA is of great significance for the development of therapies for diseases.

Due to the complex structure of GPI-TA, which consists of five subunits with multiple transmembrane domains, its structure is difficult to investigate. To date, only low-resolution structures of subunits yPIGK (24–337) [27], yPIGS (38–467) [27], and yGAA1 (170–247) [28] from *S. cerevisiae* have been observed; however, a high-resolution structure of GPI-TA has not been solved. The molecular mechanism of GPI-TA is also the most incomprehensible aspect of GPI biosynthesis. Based on the above reasons, we explored the important amino acid residues of the five subunits of GPI-TA by comparing conserved sequences and homologs, and then optimized the purification method of the GPI-TA complex for cryo-EM structure analysis. The results provide important information for acquiring a high-resolution structure of GPI-TA and understanding the molecular mechanism.

## 2. Results and Discussion

### 2.1. Exploration of Functionally Important Residues of the GPI-TA Complex

GPI-TA consists of five subunits: PIGK, GPAA1, PIGT, PIGS, and PIGU (Figure 1B). We previously constructed human embryonic kidney 293 (HEK293)-based knockout (KO) cell library defective in genes required for GPI biosynthesis [29]. The library contains KO cells defective in each GPI-TA subunit gene (*PIGK*-KO, *GPAA1*-KO, *PIGT*-KO, *PIGS*-KO, and *PIGU*-KO). To clarify the functionally important residues of each subunit, the KO cells were used to analyze the rescue of GPI-APs (CD55 and CD59) expression by transfection of the mutant constructs.

**Figure 2:**
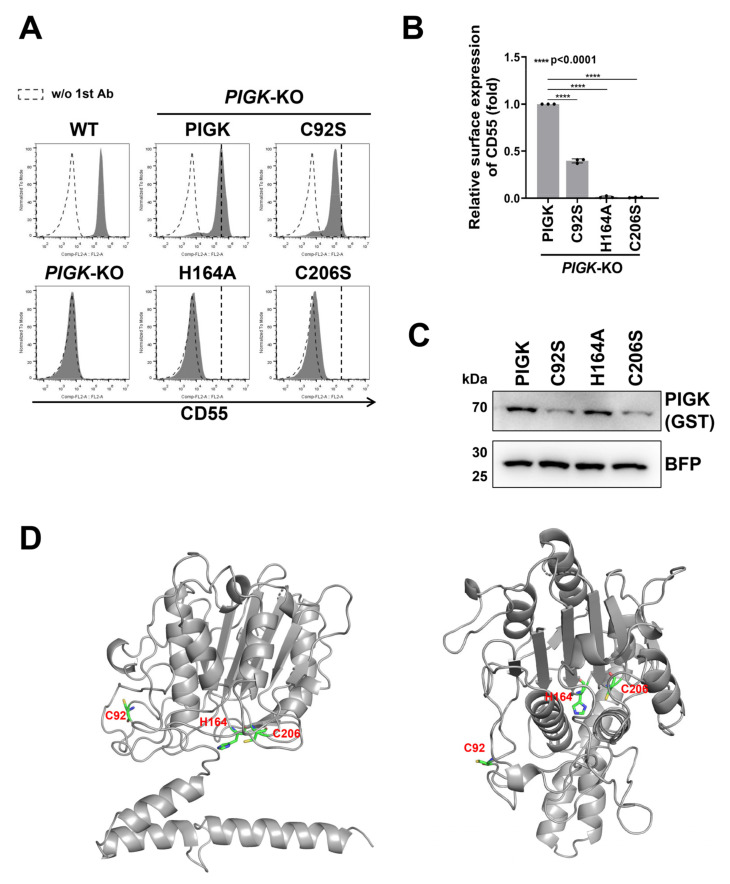
Functional analysis of mutant PIGK. (**A**) *PIGK*-KO cells were transiently transfected with both a plasmid expressing mutant PIGK and a plasmid expressing BFP. The BFP-positive cells were gated, and the surface expression of CD55 was analyzed by flow cytometry. (**B**) Mean fluorescence intensity of CD55 in *PIGK*-KO cells expressing wild-type PIGK-StrepII-GST was set to 1, and the relative intensities of CD55 in *PIGK*-KO cells expressing mutant PIGK-StrepII-GST are displayed as the mean ± SD from three independent experiments with *p*-values (unpaired Student’s *t*-test). ****, *p* < 0.0001. (**C**) Cell lysates prepared from the cells used in (**A**) were analyzed by western blotting. Expression of wild-type or mutant PIGK-StrepII-GST was detected. BFP was used as a control to check the transfection levels and loading. (**D**) PIGK homology model generated using the Phyre2 website. The functionally important residues (H164, C206, and C92) are depicted as green sticks, oxygen is shown in red, nitrogen is shown in blue, and sulfur is shown in yellow.

PIGK is the catalytic subunit of GPI-TA and shares the sequence with members of the cysteine protease family. Residues His164 and Cys206 are the putative active sites of PIGK [30], and they are highly conserved in the cysteine protease family such as legumain (UniProtKB: Q99538) [31], peptide asparaginyl ligases (UniProtKB: A0A509GV09) [32], and vacuolar-processing enzyme beta-isozyme (UniProtKB: Q39044) [33]. We expressed the H164A or C206S mutant PIGK construct in *PIGK*-KO cells. The H164A and C206S mutants did not restore the surface expression of CD55 and CD59 (Figure 2A–C and Figure S1A), indicating that the two residues were critical for GPI-TA activity. It has been reported that residue Cys92 of PIGK forms a disulfide bond with residue Cys182 of PIGT [14]. Consistent with previous results, the cell surface expression of CD55 and CD59 was decreased by 40% and 22%, respectively, in *PIGK*-KO cells expressing the PIGK C92S mutant compared with those expressing the wild-type PIGK (Figure 2A,B and Figure S1A), suggesting that the destruction of this disulfide bond reduces the activity of GPI-TA. Alternatively, the intermolecular disulfide bond would be important for the stability of PIGK, because the mutant protein levels were decreased (Figure 2C).

We next compared the *PIGK* sequences among *Homo sapiens* (UniProtKB: Q92643), *Mus musculus* (UniProtKB: Q9CXY9), *Xenopus tropicalis* (UniProtKB: Q05AQ6), *Danio rerio* (UniProtKB: Q6IQM5), *Drosophila melanogaster* (UniProtKB: Q8T4E1), and *Saccharomyces cerevisiae* (UniProtKB: P49018) to identify the important residues of PIGK (Figure S1B). Moreover, we constructed the PIGK homology model using the Phyre2 website [34] (Figure 2D). Human PIGK structure was most similar to a cysteine protease legumain [31]. The residues that were charged and conserved among species and residues around the active center of the homology model were selected and replaced with Ala. We prepared 21 mutant PIGK constructs by site-directed mutagenesis, and the rescue efficiency of GPI-TA activity in *PIGK*-KO cells was analyzed by transient transfection of the constructs (Figure S1C). Unexpectedly, although 21 mutant constructs were transfected, all the constructs rescued the expression of CD55. However, when we expressed the PIGK mutants under a minimum promoter, some mutants such as N58A, R60A, and D204A weakened the rescue efficiency compared with wild-type PIGK (Figure S1D). These results suggest that once PIGK is incorporated into the complex, it could remain active, even the activity is weakened with certain mutations, but not catalytic residue mutations.

**Figure 3:**
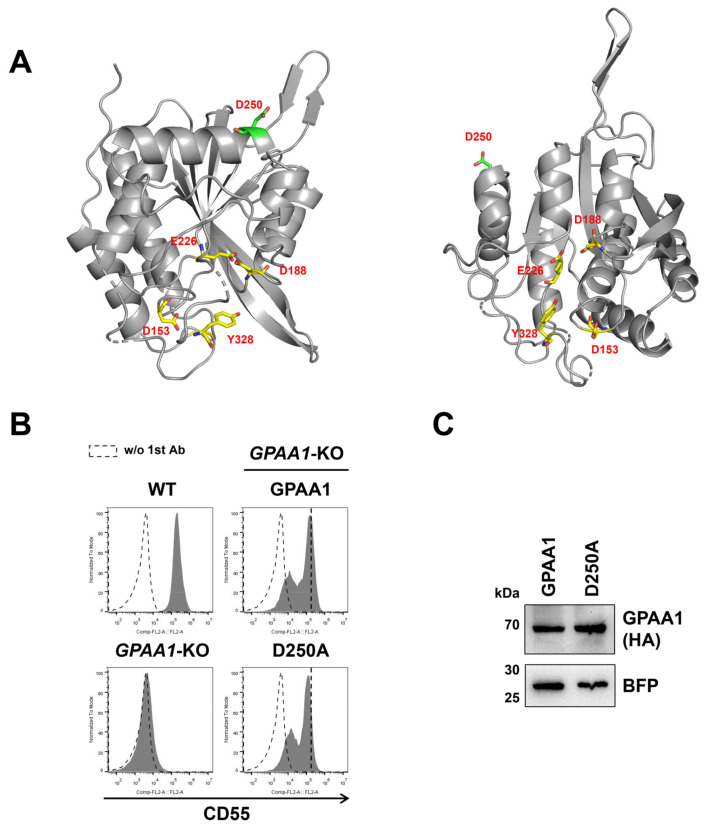
Functionally important residues of GPAA1. (**A**) GPAA1 homology model constructed using the Phyre2 website. The D250 residue is depicted in green, and the residues for putative metal binding sites are depicted in yellow. (**B**,**C**) *GPAA1*-KO cells were transiently transfected with both a plasmid expressing GPAA1-3HA (wild-type or D250A) and a plasmid expressing BFP. The BFP-positive cells were gated, and the surface expression of CD55 was analyzed by flow cytometry (**B**). Protein levels of GPAA1-3HA were detected by western blotting (**C**). BFP was used as a control to check the transfection levels and loading.

GPAA1 has weak homology to M28 family peptidases, which usually have one or two metal ions in tetrahedral coordination [35,36]. The GPAA1 homology model was constructed by Phyre2 (Figure 3A). GPAA1 was structurally similar to type II glutaminyl cyclases [37], which also belong to M28 family peptidases. Mammalian and insect type II glutaminyl cyclases were originally believed to be Zn-dependent enzymes [38,39,40]. However, several lines of evidence suggest that Zn-binding is dispensable for the activity [41,42,43,44,45]. In particular, structural analysis of glutaminyl cyclase from the black-legged tick *Ixodes scapularis* indicates that it possesses Zn ions, but that are independent of the activity [37]. For GPAA1, it is not clear whether the metal ion bindings are required for the catalytic functions. Based on the structure of the M28 peptidases, it has been reported that Asp153 and Glu226 in human GPAA1 (UniProtKB: O43292) may be residues for Zn1, and Asp153 (shared in two zinc), Asp188, and Tyr328 may be residues for Zn2 [13,16]. The Asp153, Glu226, Asp188, and Tyr328 residues are closely accommodated in the cavity of the GPAA1 structure (Figure 3A). Single point mutants (D153A, E226A, D188A, and Y328A) and double point mutants (D188A, E226A) of these residues for the putative metal binding sites could restore the CD55 expression of *GPAA1*-KO cells (Figure S2A,B). It should be noted that the rescue efficiencies of those mutant constructs by transient expression were a little weaker than that of wild-type GPAA1. These results suggest that at least these metal binding sites are not essential for GPI-TA activity, which is consistent with the independence of Zn ions for a GPAA1 structural homolog protein, *I. scapularis* glutaminyl cyclase [37] and similar catalytic mechanisms might be considered. Instead, the mutation in the Asp250 residue of GPAA1 (D250A) among the conserved sequences could not fully rescue CD55 expression (Figure S2). To confirm this finding, *GPAA1*-KO cells were cotransfected with wild-type GPAA1 or the D250A mutant GPAA1 together with a BFP-expressing construct. The BFP-positive region was gated, and the expression of CD55 and CD59 was analyzed. The D250A construct reduced GPI-TA activity without changing the protein stability (Figure 3B,C and Figure S3). In addition, mutations in N-glycosylation sites (N203Q and N517Q) of GPAA1 did not reduce the expression of CD55 on the cell surface (Figure S2).

**Figure 4:**
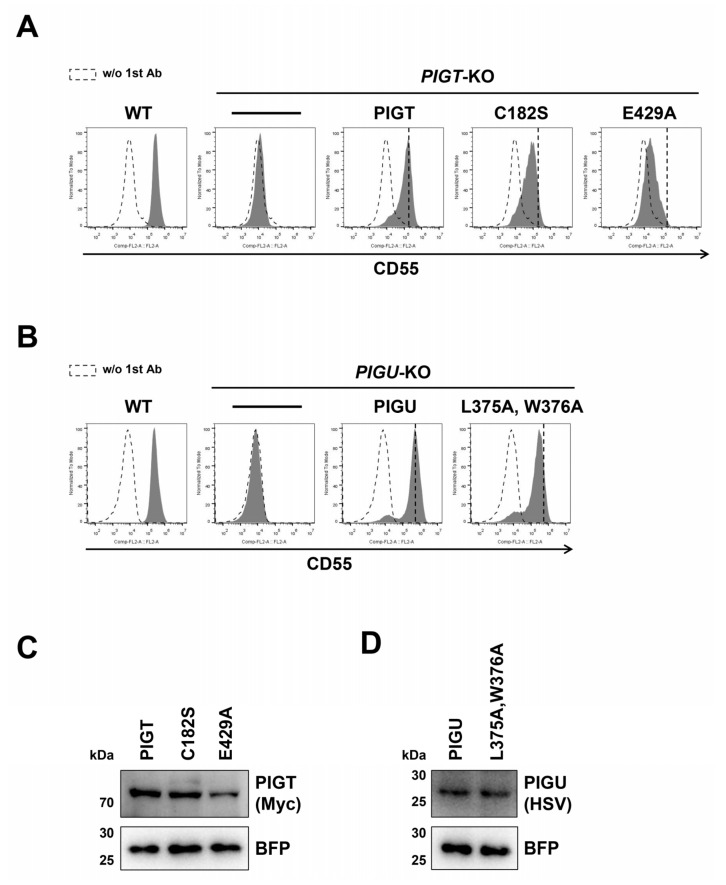
Functionally important residues of PIGT and PIGU. (**A**,**B**) Surface expression of CD55 in *PIGT*-KO (**A**) and *PIGU*-KO (**B**) cells transiently expressing wild-type or mutant PIGT-6Myc and PIGU-3HSV constructs, respectively, was analyzed by flow cytometry, as described in Figure 2A. (**C**,**D**) Expression of PIGT-6Myc (**C**) and PIGU-3HSV (**D**) mutants was detected by western blotting. BFP was used as a control to check the transfection levels and loading.

The PIGT Cys182 residue forms an intermolecular disulfide bond with C92 on PIGK, as described above [14]. The C182S mutant reduced the restoration efficiency of GPI-TA activity in *PIGT*-KO cells, which is consistent with the result of the PIGK C92S mutant (Figure 4A and Figure S3). We also checked the restoration of CD55 surface expression using constructs mutated in conserved amino acids of PIGT (Figure S4). The mutation in Glu429 of PIGT (E429A) lost GPI-TA activity (Figure 4A and Figure S3). The expression of E429A was reduced compared to that of the wild-type Myc-PIGT (Figure 4C), suggesting that Glu429 is required for PIGT stability or assembly into the GPI-TA complex. In addition, the conserved amino acids in PIGS among species were mutated and the restoration of the activity was analyzed using KO cells (Figure S5). To analyze the importance of N-glycosylation sites on each subunit protein, N-glycosylation sites were mutated in PIGT (N164Q, N291Q, and N327Q) (Figure S4) and PIGS (N267Q and N370Q) [21] (Figure S5). All the constructs mutated in N-glycosylation sites could restore the expression of CD55 on the cell surface, suggesting that N-glycans on the subunits do not affect GPI-TA activity. Among the PIGU constructs mutated in the conserved amino acids (Figure S6), the construct with mutations in both Leu375 and Trp376 residues (L375A, W376A) reduced the restoration activity (Figure 4B,D and Figure S3). The protein expression level of the L375A and W376A mutants was the same as that of the wild-type HSV-PIGU. Taken together, we explored the functionally important residues of the GPI-TA complex. In addition to the catalytic active sites on PIGK (His164 and Cys206) and Cys residues that form an intermolecular disulfide bond between PIGK (Cys92) and PIGT (Cys182), we found important residues for GPI-TA activity including GPAA1 (Asp250), PIGT (Glu429), and PIGU (Leu375, Phe376).

### 2.2. Expression of PIGK Recombinant Protein

**Figure 5:**
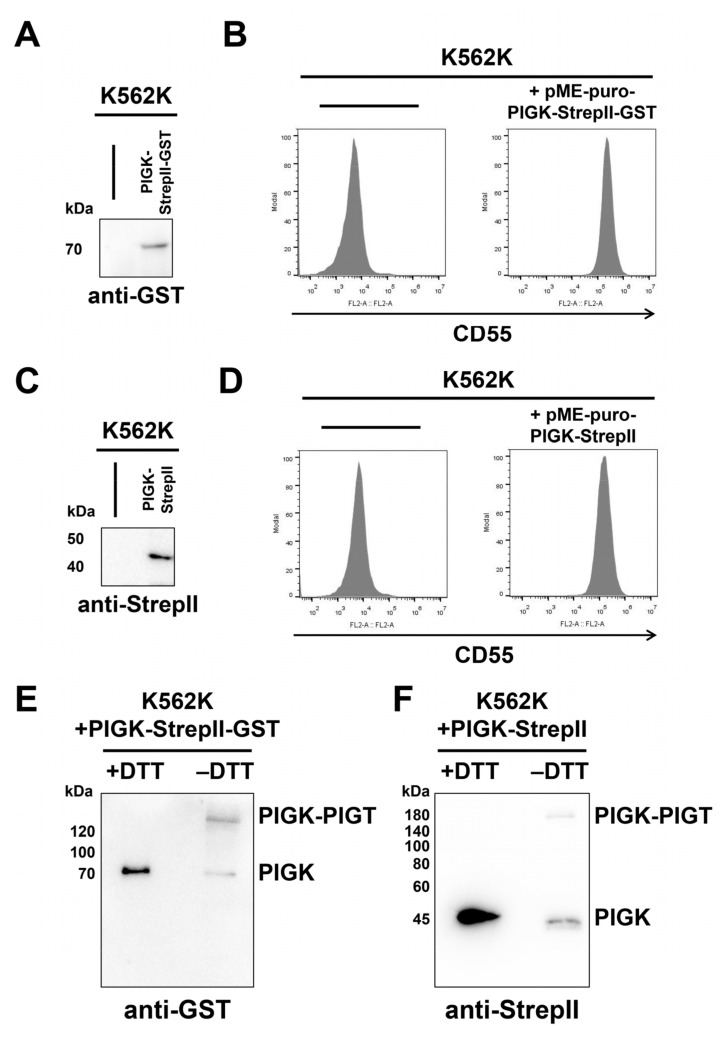
Establishment of cells for GPI-TA purification. (**A**,**B**) K562 *PIGK*-KO (K562K) cells and K562K cells stably expressing PIGK-StrepII-GST were lysed, and PIGK-StrepII-GST was detected by the anti-GST antibody (**A**). The surface expression of CD55 in these cells was analyzed using flow cytometry (**B**). (**C**,**D**) K562 *PIGK*-KO (K562K) cells and K562K cells stably expressing PIGK-StrepII were lysed, and PIGK-StrepII was detected by the anti-StrepII antibody (**C**). Surface expression of CD55 in those cells was analyzed using flow cytometry (**D**). (**E**,**F**) PIGK-StrepII-GST (**E**) and PIGK-StrepII (**F**) samples were prepared under reducing (sample buffer with dithiothreitol (+DTT)) and nonreducing (sample buffer without dithiothreitol (−DTT)) conditions. Both PIGK-StrepII-GST and PIGK-StrepII were detected at higher molecular weights under the nonreducing condition, indicating the formation of intermolecular disulfide bonds with PIGT.

To understand the GPI-TA structure, we next examined methods for purification of the GPI-TA complex in a large scale. StrepII-GST-tagged or StrepII-tagged human PIGK was stably expressed in *PIGK*-deficient human K562 (K562K) cells [46]. The expression of hPIGK-StrepII-GST and hPIGK-StrepII was detected at 70 kDa by anti-GST (Figure 5A) and 45 kDa by anti-StrepII (Figure 5C), respectively. Flow cytometric analysis confirmed that both constructs successfully rescued the expression of CD55 on the cell surface in K562K cells (Figure 5B,D). Under nonreducing conditions (-DTT), both recombinant hPIGK-StrepII-GST and hPIGK-StrepII migrated slowly and were detected at higher molecular weights (Figure 5E,F), suggesting the formation of an intermolecular disulfide bond with PIGT. These results support that both recombinant hPIGK-StrepII-GST and hPIGK-StrepII form active GPI-TA complexes with PIGT, PIGU, PIGS, and GPAA1.

### 2.3. Optimization of the Purification Conditions of the GPI-TA Complex

Since a GPI-TA complex has ~20 transmembrane domains in total (Figure 1B), it is difficult to purify GPI-TA without destroying the complex structure. First, we tried to use StrepTactin Sepharose and Glutathione Sepharose beads to purify the GPI-TA complex. However, the reduced glutathione contained in the elution buffer of Glutathione Sepharose beads broke the disulfide bond between PIGK and PIGT (Figure S7A). At the same time, β-mercaptoethanol (β-ME), a reducing reagent commonly used in purification experiments at low concentrations, also destroyed the structure of the GPI-TA complex (Figure S7B). Therefore, we chose to use StrepTactin Sepharose beads for single-step purification of the GPI-TA complex without adding any reducing reagent.

The choice of detergent is critical for the solubilization, monodispersity, and stability of membrane proteins in the purification process. In addition, detergent affects the dispersal of protein samples on the grid for cryogenic electron microscopy (cryo-EM) analysis. First, we applied three kinds of detergent to purify the GPI-TA complex: n-dodecyl-β-D-maltopyranoside (DDM); cholesteryl hemisuccinate (CHS), which was added to DDM in a ratio of 1:5 (*w*:*w*), with the resulting detergent called DDM-CHS; and digitonin. Among them, the lipid compound CHS was used in the purification and crystallization of various GPCRs [47,48,49], thus stabilizing membrane proteins [50]. In the purified protein fraction, all five subunits of the GPI-TA complex could be detected by silver staining and were confirmed by mass spectrometry (Figure S7C and Table S1), and part of the disulfide bonds between PIGK and PIGT were retained under conditions using any of the detergents (Figure S7D). However, when DDM was used as a detergent, the purified protein structure was unstable and the protein mass fraction was decreased when analyzed by size exclusion chromatography (SEC) (Figure S7E). The cryo-EM results showed that the protein particles purified with DDM-CHS were scattered on the edges because the ice in the center was too thin. The particles in the center were evenly dispersed (Figure S7F). Compared to DDM-CHS, digitonin particles have more difficulty entering the holes in the samples. When using DDM, only small particles were observed, which may be individual subunits of the GPI-TA complex.

**Figure 6:**
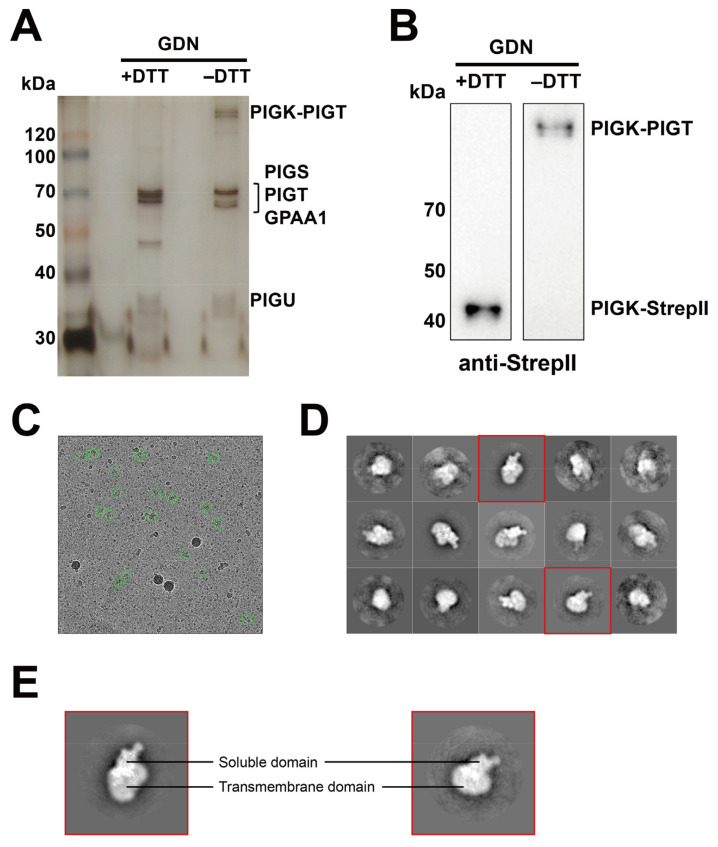
Large-scale purification of the GPI-TA complex by GDN. (**A**,**B**) PIGK-StrepII containing the GPI-TA complex was purified in buffer containing GDN. The purified samples were detected by silver staining (**A**) or by western blotting (**B**). Samples were detected under reducing (+DTT) or nonreducing (−DTT) conditions as described in Figure 5. For western blotting, an anti-StrepII antibody was used to detect PIGK-StrepII. (**C**) EM images of the purified PIGK-StrepII containing the GPI-TA complex in GDN, which were obtained using a 300 kV cryo-EM equipped with a K2 summit direct electron detector camera. (**D**) Representative 2D classification of cryo-EM data showing different orientations of the protein, with the box size of 390 angstrom and the mask of 300 angstrom processed on RELION. Red square, selected images used in (**E**). (**E**) Particle features observed from the 2D classes. Putative soluble domain and transmembrane domain are indicated, respectively.

We next applied glyco-diosgenin (GDN) as a detergent during purification. Since it has multiple advantages such as >10% solubility and nontoxic byproducts, it is widely used to solubilize and purify membrane proteins for the purpose of structural and cryo-EM studies [51,52,53,54]. In addition, we chose K562K cells expressing hPIGK-StrepII instead of hPIGK-GST-StrepII to purify the GPI-TA complex since the GST tag is relatively large and easily forms dimer structures [55]. Membrane fraction was first solubilized in solubilization buffer containing DDM-CHS, then the soluble fraction was purified in buffer containing GDN. The purified GPI-TA complex containing all five subunits could be observed (Figure 6A). Most of the PIGK formed disulfide bonds with PIGT under nonreducing conditions (Figure 6A,B). In addition, the protein particles with GDN were equally dispersed on the grids for cryo-EM (Figure 6C) compared to the other detergents. Therefore, we chose K562K-hPIGK-StrepII cells and GDN as detergents for purification of the GPI-TA complex.

### 2.4. Preliminary Cryo-EM Analysis of GPI-TA Complex

To date, low-resolution structures of yPIGK (24–337), yPIGS (38–467) [27], and yGAA1 (170–247) [28] from *S. cerevisiae* have been resolved. However, little is known about the structure of the GPI-TA complex. Using the GDN-purified GPI-TA complex containing hPIGK-StrepII, we performed a preliminary cryo-EM on the GPI-TA complex to evaluate the sample quality. Single particle analysis of the GPI-TA complex using 300 kV cryo-EM equipped with a K2 summit direct electron detector camera showed monodisperse particles (Figure 6C). The particles extracted from more than two thousand images were classified into several representative 2D classes (Figure 6D). At this resolution, it was difficult to verify the stoichiometry and composition of the GPI-TA complex, whereas putative transmembrane regions and a soluble globular domain could be observed (Figure 6E). The soluble domain would be the luminal domain of GPI-TA based on the protein topology and the active site. These results indicate the sample quality of GDN-purified GPI-TA, which would be suitable for structural studies. By using the proposed method and selecting more high-quality single particles, the structure of the fully assembled GPI-TA complex could be acquired in future analysis.

## 3. Conclusions

In this study, we analyzed the roles of amino acid residues of the GPI-TA subunits in the activity. In addition to catalytic residues (His164 and Cys206) of PIGK and residues for the intermolecular disulfide bond between PIGK (Cys92) and PIGT (Cys182), we found several amino acid residues (Asp250 on GPAA1, Glu429 on PIGT, Leu375 and Phe376 on PIGU), which are critical for GPI-TA activity or stability. We further optimized the purification process of the GPI-TA and applied the purified complex for a preliminary structural analysis using single-particle imaging. We found that solubilization of GPI-TA in buffer containing DDM-CHS detergent, followed by purification in buffer containing GDN detergent was the best for keeping five subunits and particle distribution on the grids for cryo-EM. Our study provides the basis for functional and structural analysis of the GPI-TA complex.

## 4. Materials and Methods

### 4.1. Cell Lines, Antibodies, and Reagents

HEK293 (ATCC CRL-1573) cells and their derivatives were cultured in Dulbecco’s modified Eagle medium (DMEM) with high glucose, glutamine (01-052-1ACS, Biological Industries, Beit HaEmek, Israel) and 10% fetal bovine serum (04-001-1ACS, Biological Industries, Israel) at 37 °C in a humidified 5% CO_2_ atmosphere. *PIGK*-KO, *GPAA1*-KO, *PIGT*-KO, *PIGS*-KO, and *PIGU*-KO cells were previously constructed [29]. K562K (K562 *PIGK*-KO) [46] cells were cultured in DMEM with high glucose, glutamine and 10% fetal bovine serum. K562K-hPIGK-StrepII or K562K-hPIGK-StrepII-GST cells were established by transfection of the plasmid pME-puro-hPIGK-StrepII or pME-puro-hPIGK-StrepII-GST into K562K cells, respectively, and selection using puromycin (ant-pr-5, In vivo Gen, San Diego, CA, USA).

Mouse monoclonal anti-CD55 (clone IA10) [56], anti-CD59 (clone 5H8) [57], anti-GST (HT601, TransGen Biotech, Beijing, China), anti-Myc (HT101, TransGen Biotech), anti-StrepII (M211-3, MBL, Nagoya, Japan), rabbit monoclonal anti-HA (3724, Cell Signaling Technology, Danvers, MA, USA), anti-RFP (R10367, Thermo Fisher Scientific, Waltham, MA, USA), and anti-HSV (BRP0002, Beijing B&M Biotech, Beijing, China) were used as primary antibodies. F(ab’)2-goat anti-mouse IgG (H + L) PE (12-4010-82; Thermo Fisher Scientific) (for flow cytometric analysis), goat anti-mouse IgG (H + L) HRP (HS201, TransGen Biotech), and goat anti-rabbit IgG (H + L) HRP (HS101, TransGen Biotech) (for western blotting) were used as the secondary antibodies. StrepTactin Sepharose (28-9355-99, GE, Chicago, IL, USA) was used to purify the GPI-TA.

### 4.2. Plasmids

All primers used in this study are listed in Table S2. The DNA fragments corresponding to human PIGK were amplified from pPB-FRT-hPIGK. The HRV3C protease cleavage site and StrepII were amplified from the forward primer Strep-tag-F and the reverse primer Strep-tag-R. The DNA fragments consisting of hPIGK and StrepII (containing the HRV3C protease cleavage site) were ligated into the EcoRI/NotI site of the vector pME-puro to generate pME-puro-hPIGK-StrepII by using the M-Quick Cloning Mix (T1406, Talen-bio, Shanghai, China). The DNA fragments corresponding to GST were amplified from pME-GST and ligated into the SalI/NotI site of the vector pME-puro-hPIGK-StrepII to generate pME-puro-hPIGK-StrepII-GST.

The DNA fragments corresponding to hGPAA1-3HA, hPIGT-6Myc, FLAG-hPIGS, and hPIGU-3HSV, which were amplified from pME-hGPAA1-3HA, pME-hPIGT-6Myc, pME-FLAG-hPIGS, and pME-hPIGU-3HSV, respectively, were ligated into the EcoRI/NotI site of the vector pME-Hyg to generate pME-Hyg-hGPAA1-3HA, pME-Hyg-hPIGT-6Myc, pME-Hyg-FLAG-hPIGS, and pME-Hyg-hPIGU-3HSV.

Plasmids harboring mutant *PIGK*, *GPAA1*, *PIGT*, *PIGS*, or *PIGU* were constructed by site-direct mutagenesis based on pME-puro-hPIGK-StrepII-GST, pME-Hyg-hGPAA1-3HA, pME-Hyg-hPIGT-6Myc, pME-Hyg-FLAG-hPIGS, or pME-Hyg-hPIGU-3HSV, respectively. Generally, conserved amino acids were replaced with Ala or Ser, and the Asn residues on N-glycosylation sites were mutated to Gln. The primers used for the construction of mutant plasmids are listed in Table S2.

### 4.3. Transient Transfection and Flow Cytometric Analysis

For transient transfection to screen various mutant constructs, cells (~10^6^ cells/well) were plated in 6-well plates one day before transfection. Four micrograms of plasmids were transfected into KO cells using polyethyleneimine MAX (PEI-MAX) (24765, Polysciences, Warrington, PA, USA) in OPTI-MEM (31985062, Thermo Fisher Scientific). Three days after transfection, the transfected cells were harvested and washed with PBS (E607009, Sangon Biotech, Shanghai, China). Then, the samples were stained with primary antibodies (10 µg/mL) in FACS buffer (PBS containing 1% BSA and 0.1% NaN3) for 25 min on ice. After washing twice with FACS buffer, the samples were stained with the secondary antibody F(ab’)2-goat anti-mouse IgG (H + L) PE (10 µg/mL) in FACS buffer for 25 min on ice. The samples were then washed twice with FACS buffer and analyzed using an Accuri C6 system (BD). Accuri C6 and FlowJo software (BD) were used to analysis the data.

For co-transfection with a BFP construct, cells (~5 × 10^6^ cells/well) were plated in 12-well plates one day before transfection. Plasmids (0.8 μg of plasmids expressing GPI-TA genes and 0.8 μg of plasmids expressing BFP (pME-tagBFP)) were mixed and transfected into KO cells using PEI-MAX. Three days after transfection, the transfected cells were harvested and stained with antibodies as described above. In mammalian cells, co-transfection efficiency is generally high. Therefore, BFP-positive cells were gated and only plasmid-transfected cells were analyzed using an Accuri C6 system.

### 4.4. Western Blotting

Western blotting was performed to confirm the expression level of recombinant proteins. Cells (~10^6^ cells/well) were lysed with 100 μL of lysis buffer (150 mM NaCl, 50 mM Tris-HCl pH 7.5, 1% NP-40, 1 mM phenylmethylsulfonyl fluoride (PMSF) (HY-B0496, MedChemExpress, Monmouth Junction, NJ, USA), protein inhibitor cocktail (HY-K0010, MedChemExpress) on ice for 30 min. When detecting the recombinant protein PIGU-3HSV, an additional 8 M urea was added to the lysis buffer. After incubation, the sample was centrifuged at 21,600× *g* for 10 min at 4 °C to remove insoluble fractions. The supernatant was mixed with SDS-PAGE loading buffer and kept at 4 °C overnight. To check the protein levels of GPI-TA mutant proteins, BFP was used as a control to check the transfection levels and loading. A plasmid expressing BFP (pME-tagBFP) and mutant GPI-TA gene constructs at a ratio of 1:1 were mixed and co-transfected into cells. When cell lysate samples were prepared, at least BFP expression levels of cells in Figure 2C, Figure 3C and Figure 4C,D were similar among samples.

### 4.5. Stable Transfection of hPIGK-StrepII or hPIGK-StrepII-GST

Twenty micrograms of pME-puro-hPIGK-StrepII or pME-puro-hPIGK-StrepII-GST was digested by FspI (R0135s, New England BioLabs, Ipswich, MA, USA) for 4 h at 37 °C. The linearized plasmid was transfected into K562K cells (~10^7^ cells) suspended in 800 μL of OPTI-DMEM by electroporation. Cells expressing hPIGK-StrepII or hPIGK-StrepII-GST were screened using the antibiotic puromycin.

### 4.6. Purification of the GPI-TA Complex

All the purification steps were performed at 4 °C. K562K-hPIGK-StrepII cells (~50 L) were harvested by centrifugation and washed with PBS. The cells were disrupted with a microfluidizer in TBS (50 mM Tris pH 7.5, 150 mM NaCl) (with 1 mM PMSF, protein inhibitor cocktail), and the debris was removed by centrifugation (8400× *g*) for 20 min. The collected supernatant was ultracentrifuged at 100,000× *g* for 1 h to collect the membrane. Then, the membranes were solubilized by adding 150 mL of solubilization buffer (50 mM Tris pH 7.5, 150 mM NaCl, 2% n-dodecyl-β-d-maltopyranoside (DDM) (69227-93-6, Anatrace, Maumee, OH)—0.4% cholesteryl hemisuccinate (CHS) (C6512, Merck, Burlington, MA, USA), 1 mM PMSF, protein inhibitor cocktail). After incubation with rotation for 2 h, the mixture was ultracentrifuged at 100,000× *g* for 1 h to remove unsolubilized debris. The supernatant was mixed with 5 mL of StrepTactin Sepharose resin pre-equilibrated with buffer A (50 mM Tris pH 7.5, 150 mM NaCl, 0.06% glyco-diosgenin (GDN) (GDN101, Anatrace) and rotated for 1 h [FT]. StrepTactin Sepharose was collected through the column and washed 10 times with 5 mL of buffer A [W]. The GPI-TA complex was eluted by 5 mL of buffer B (buffer A containing 2.5 mM desthiobiotin (D1411, Merck)) five times [E1, E2, E3, E4, E5].

### 4.7. Sliver Staining

To detect the purity of the purified GPI-TA complex, a Fast Silver Stain Kit (P0017S, Beyotime, Shanghai, China) was used according to the instructions. The protein bands were cut, and the peptides were analyzed using mass spectrometry to verify whether GPI-TA was successfully purified.

### 4.8. EM Data Acquisition

A total of 2.5 μL of the GPI-TA complex sample was applied to a glow-discharged holey carbon-film grid (Quantifoil, Au 1.2/1.3, 300 mesh) blotted with a Vitrobot system (FEI, Hillsboro, OR) using a 3.0 s blotting time with 100% humidity at 9 °C, then the sample was plunge-frozen in liquid ethane. Cryo-EM data collection was performed using a 300 kV Titan Krios microscope (FEI) equipped with a K2 summit direct electron detector camera (Gatan Inc., Pleasanton, CA, USA) set to super resolution mode, with a pixel size of 0.65 Å (a physical pixel size of 1.3 Å) and a defocus ranging from −1.5 µm to −2.3 µm. The specimen stage temperature was maintained at 80 K. The dose rate was 10 e^−^ s^−1^, and each movie was 7.6 s long, dose-fractioned into 38 frames with an exposure of 1.18 e^−^ Å^−2^ for each frame.

### 4.9. Image Processing

A total of 2445 movies were motion-corrected and binned with MotionCor2 [58] with 5 × 5 patches, which produced summed and dose-weighted micrographs with a pixel size of 1.3 Å. Contrast transfer function (CTF) parameters were estimated by CTFFIND 4.1 [59]. Particle picking and further image processing were performed using RELION 3.0 [60]. A total of 132,558 particles were autopicked and extracted with a box size of 300 × 300 pixels. 2D classifications were then performed.

## Data Availability

The data that supports the findings of this study are available in the figures and Supplementary Materials in this article.

## Supplementary Materials

The following are available online: Figure S1. The effect of mutant PIGK on GPI-TA activity, Figure S2. The effect of mutant GPAA1 on GPI-TA activity, Figure S3. The effect of mutant PIGT on GPI-TA activity, Figure S4. The effect of mutant PIGS on GPI-TA activity, Figure S5. The effect of mutant PIGU on GPI-TA activity, Figure S6. Optimization of large-scale purification for the GPI-TA complex, Table S1. LC-MS analysis of the purified GPI-TA complex, Table S2. List of primers used in this study.

## Sample Availability

Samples generated in this study are available from the authors.
