# Supplementary material for: Functional Analysis of the GPI Transamidase Complex by Screening for Amino Acid Mutations in Each Subunit
Source: Molecules. 2021 Sep 8;26(18):5462. doi: 10.3390/molecules26185462 (PMC8465894; doi:10.3390/molecules26185462)
Supplement: Supplementary file 1 [file molecules-26-05462-s001.zip › molecules-1325274-supplementary.pdf]

Supplementary Figure S1

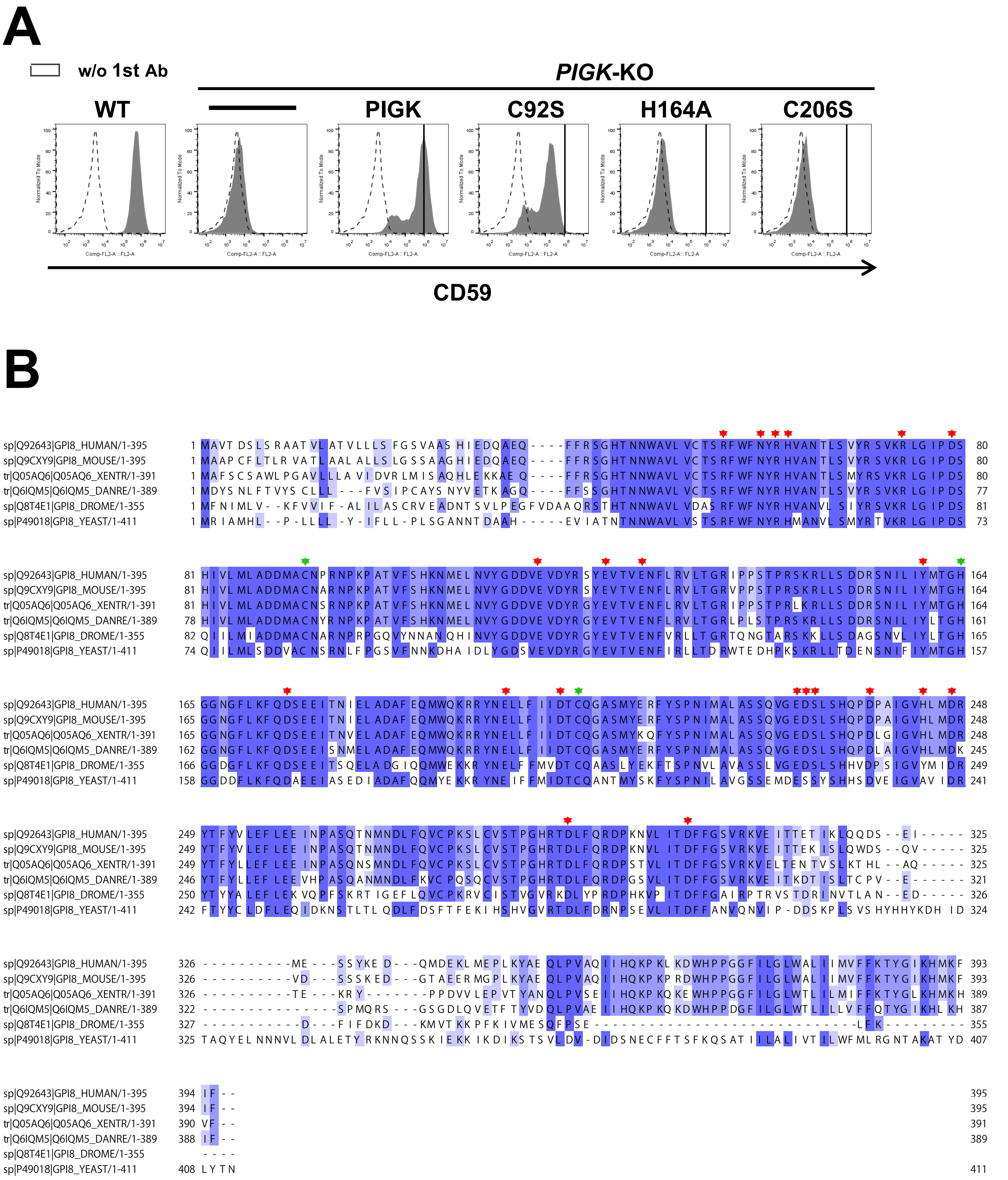

Supplementary Figure S1

C

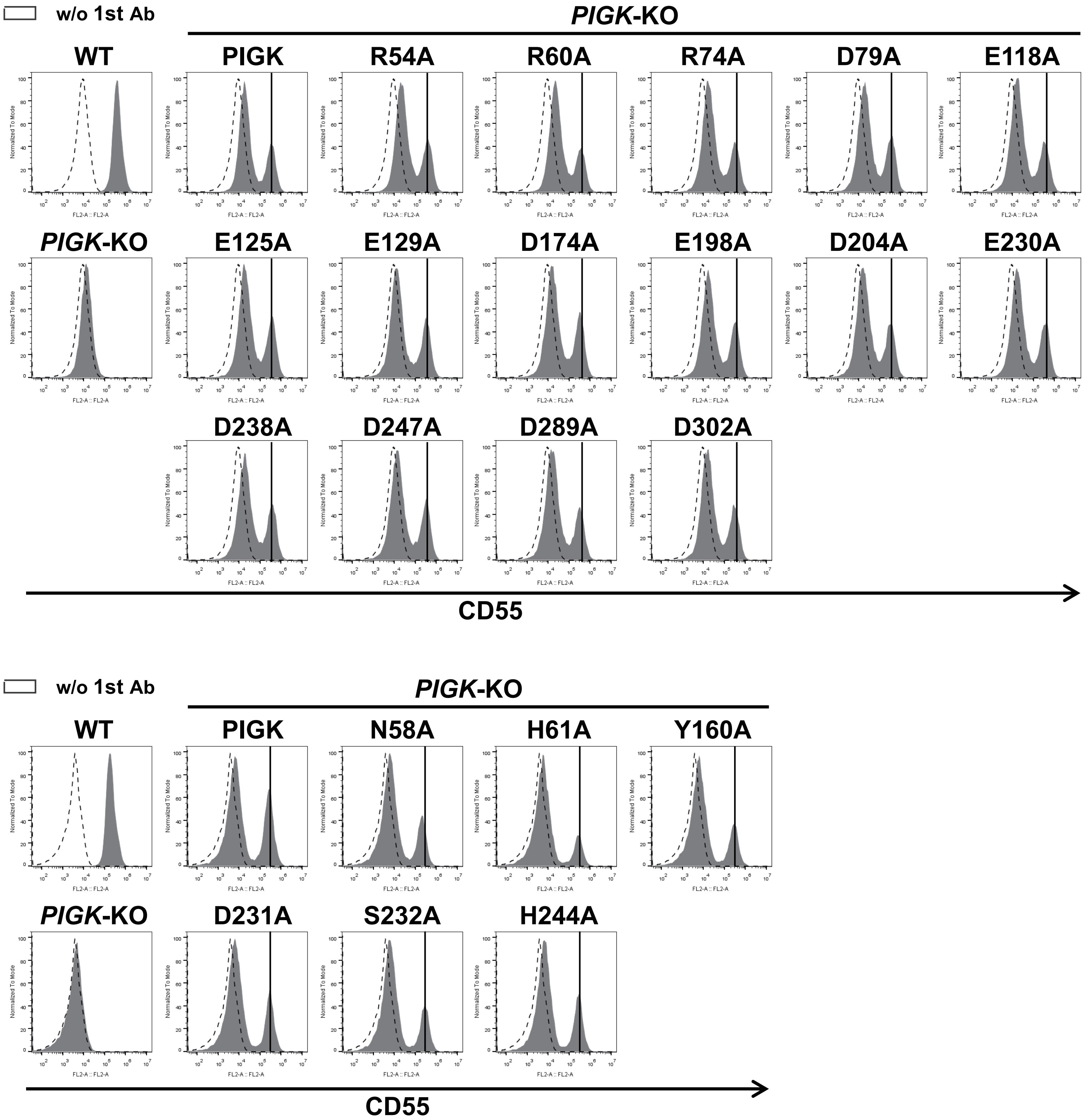

Supplementary Figure S1

D

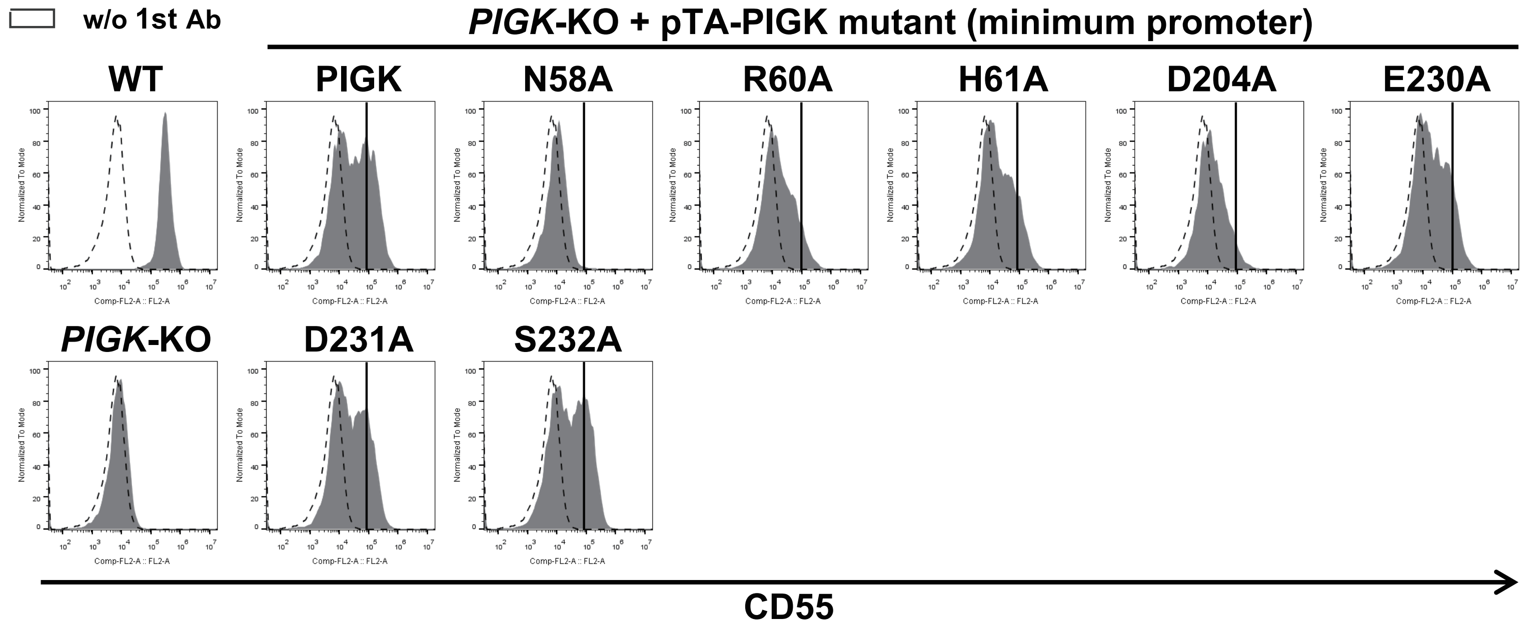

Figure S1. The effect of mutant PIGK on GPI-TA activity.

A) PIGK-KO cells were transiently transfected with both a plasmid expressing mutant PIGK and a plasmid express-ing BFP. The BFP-positive cells were gated, and the surface expression of CD59 was analyzed by flow cytometry.

B) Sequence alignment of PIGK proteins. PIGK sequences among *Homo sapiens* (UniProtKB: Q92643), *Mus mus-culus* (UniProtKB: Q9CXY9), *Xenopus tropicalis* (UniProtKB: Q05AQ6), *Danio rerio* (UniProtKB: Q6IQM5), *Dro-sophila melanogaster* (UniProtKB: Q8T4E1), and *Saccharomyces cerevisiae* (UniProtKB: P49018) are aligned. Con-served amino acids are colored in blue. Red stars indicate amino acids are mutated in this study, but are not affected in the GPI-TA function. The amino acids, whose mutations reduced the GPI-TA activity, are shown as green stars.

C) The indicated mutant PIGK constructs were transiently expressed in PIGK-KO cells. HEK293 (WT), PIGK-KO cells, and PIGK-KO cells transiently transfected with wild-type PIGK were used as controls. Surface expression of CD55 was detected using flow cytometry.

D) PIGK-KO cells were transiently transfected with both a plasmid expressing PIGK mutants under a minimum promotor (pTA) and a plasmid expressing BFP. The BFP-positive cells were gated, and the surface expression of CD55 was analyzed by flow cytometry.

Supplementary Figure S2

A

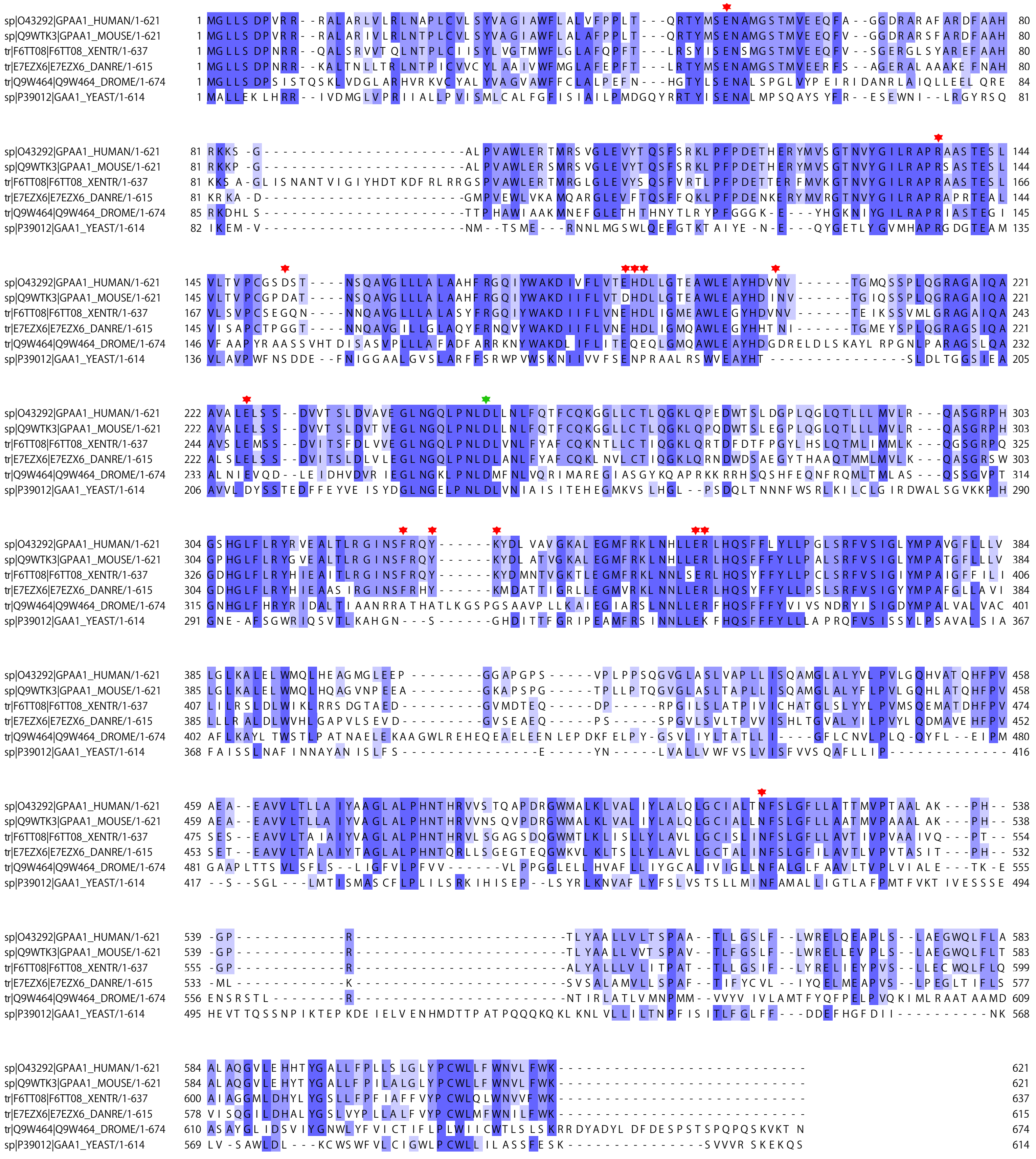

Supplementary Figure S2

B

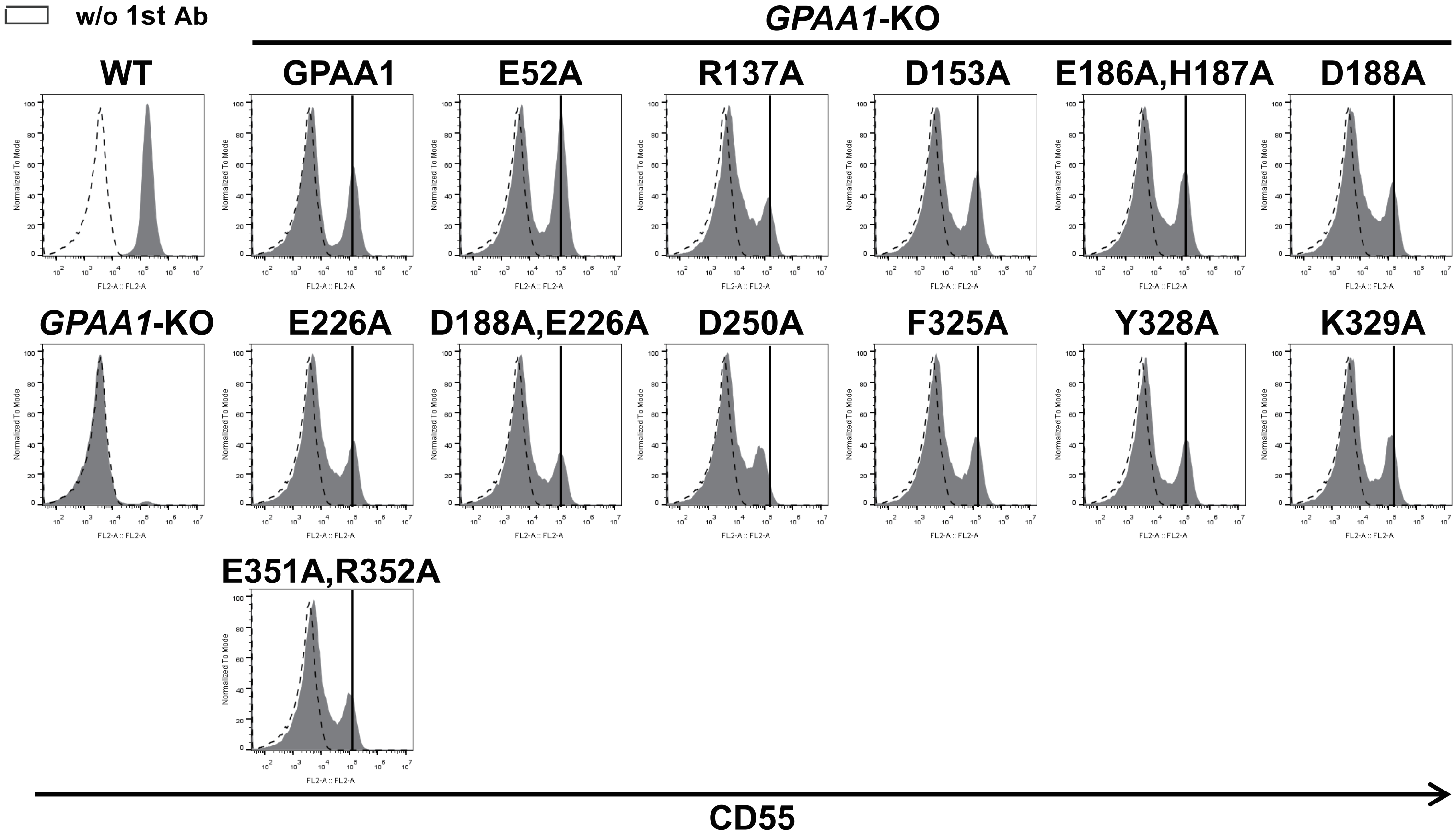

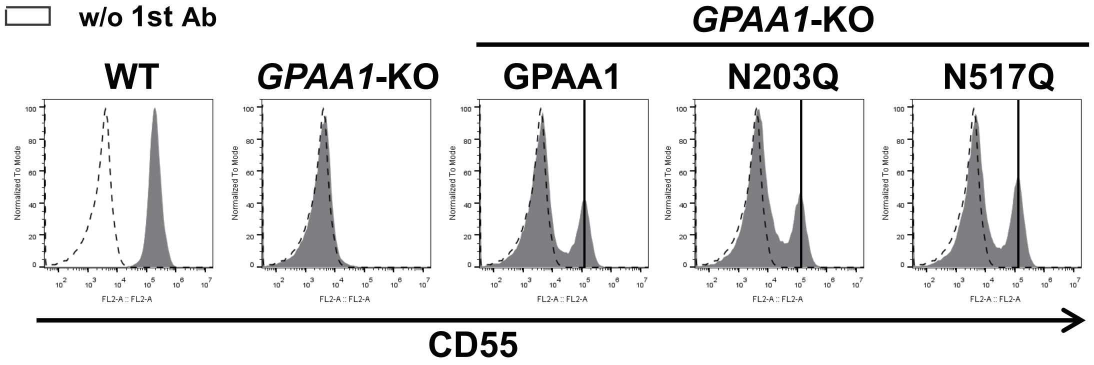

Figure S2. The effect of mutant GPAA1 on GPI-TA activity.

A) Sequence alignment of GPAA1 proteins. GPAA1 sequences among *H. sapiens* (UniProtKB: O43292), *M. musculus* (UniProtKB: Q9WTK3), *X. tropicalis* (UniProtKB: F6TT08), *D. rerio* (UniProtKB: E7EZX6), *D. melanogaster* UniProtKB: (Q9W464), and *S. cerevisiae* (UniProtKB: P39012) are aligned. Conserved amino acids are colored in blue. Red stars indicate amino acids are mutated in this study, but are not affected in the GPI-TA function. The amino acid, whose mutation reduced the GPI-TA activity, is shown as a green star.

B) The indicated mutant GPAA1 constructs were transiently expressed in *GPAA1*-KO cells. HEK293 (WT), *GPAA1*-KO cells, and *GPAA1*-KO cells transiently transfected with wild-type GPAA1 were used as controls. Surface expression of CD55 was detected using flow cytometry. In addition to the mutants in the conserved residues of GPAA1, mutations of the N-glycosylation sites were detected.

Supplementary Figure S3

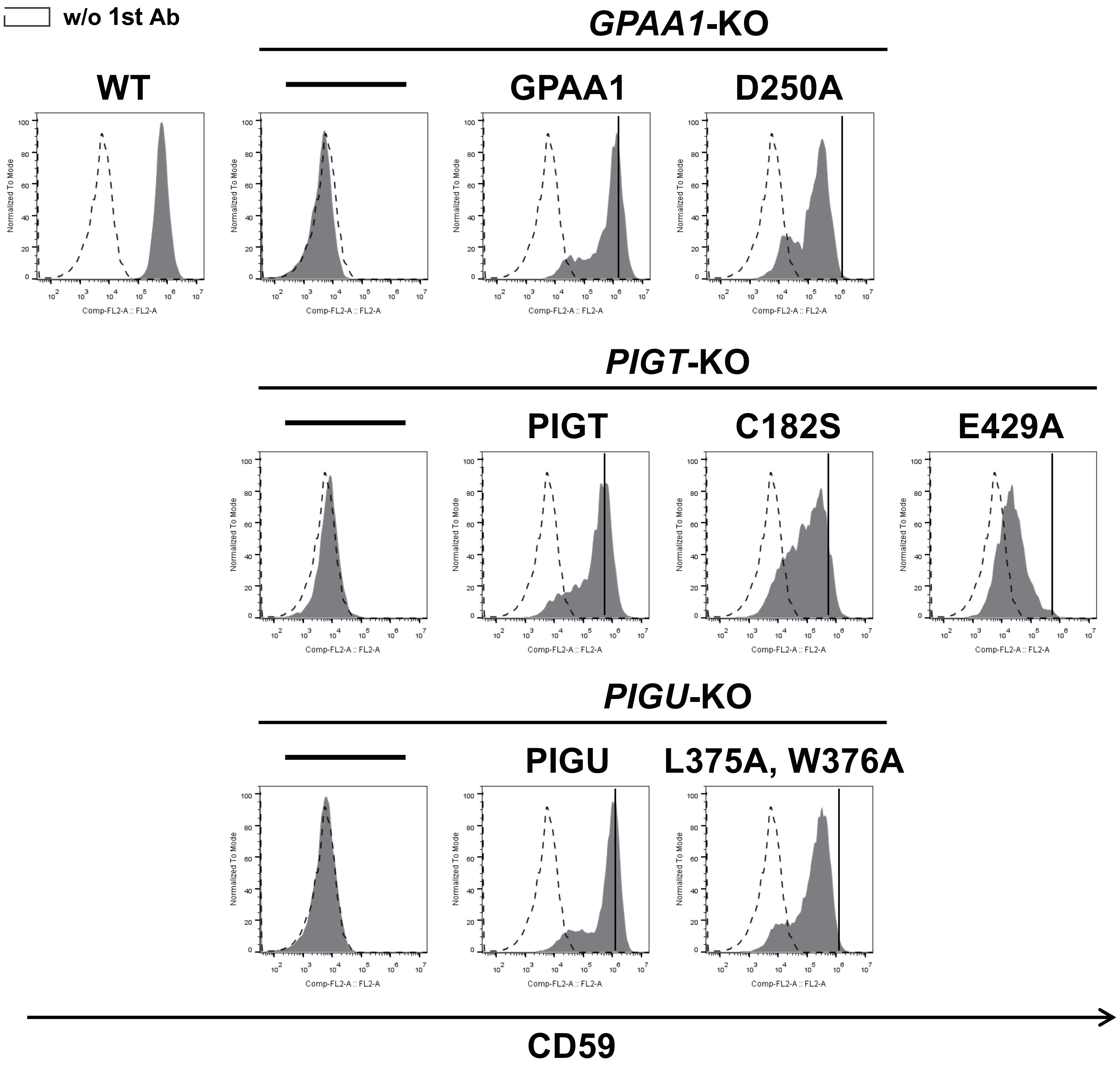

Figure S3. Functionally important residues of GPAA1, PIGU, and PIGT.
Surface expression of CD59 in GPAA1-KO, PIGT-KO, and PIGU-KO cells transiently expressing wild-type or mutant GPAA1-3HA, PIGT-6Myc, and PIGU-3HSV constructs, respectively, was analyzed by flow cytometry as described in Figure 2A.

Supplementary Figure S4

A

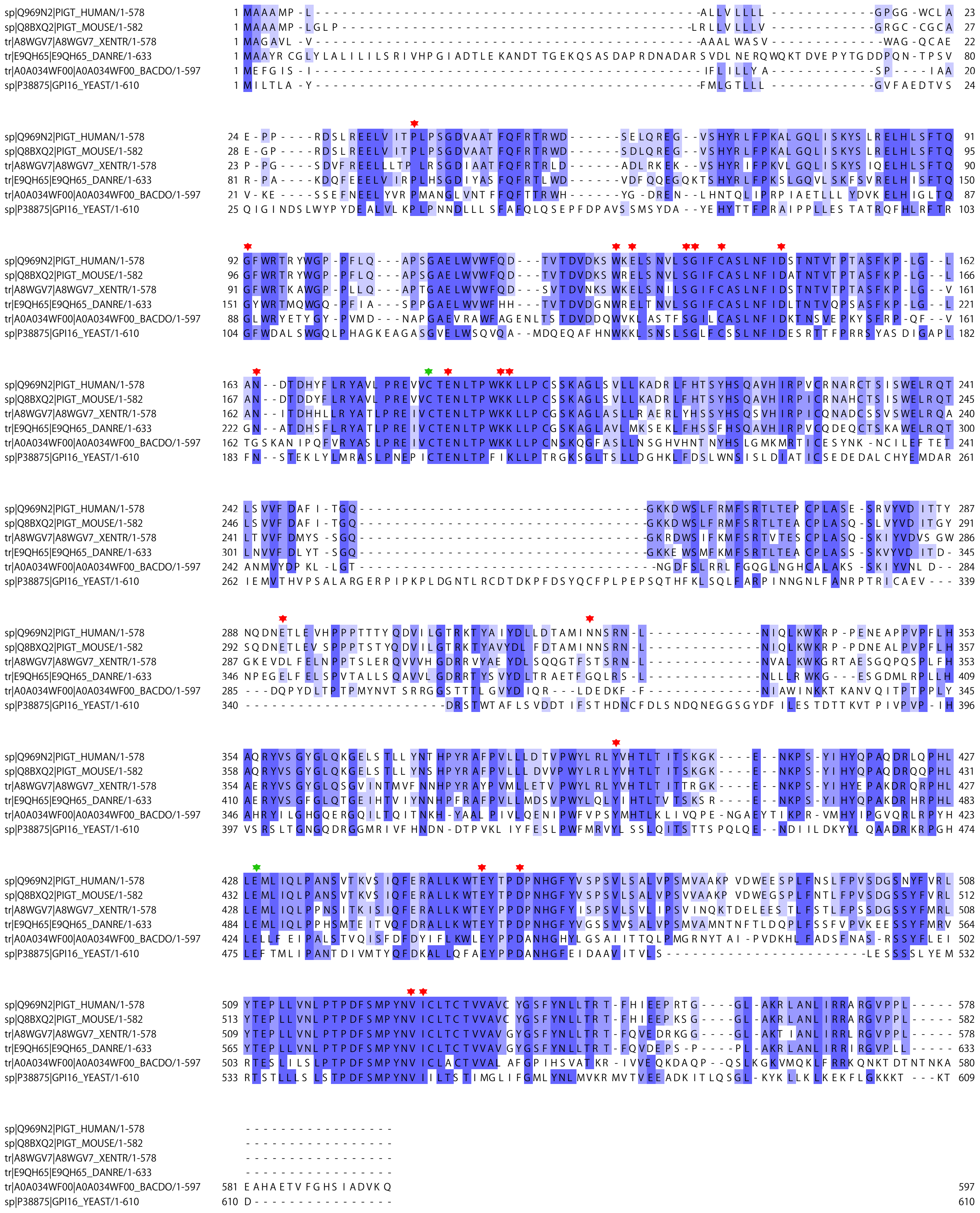

Supplementary Figure S4

B

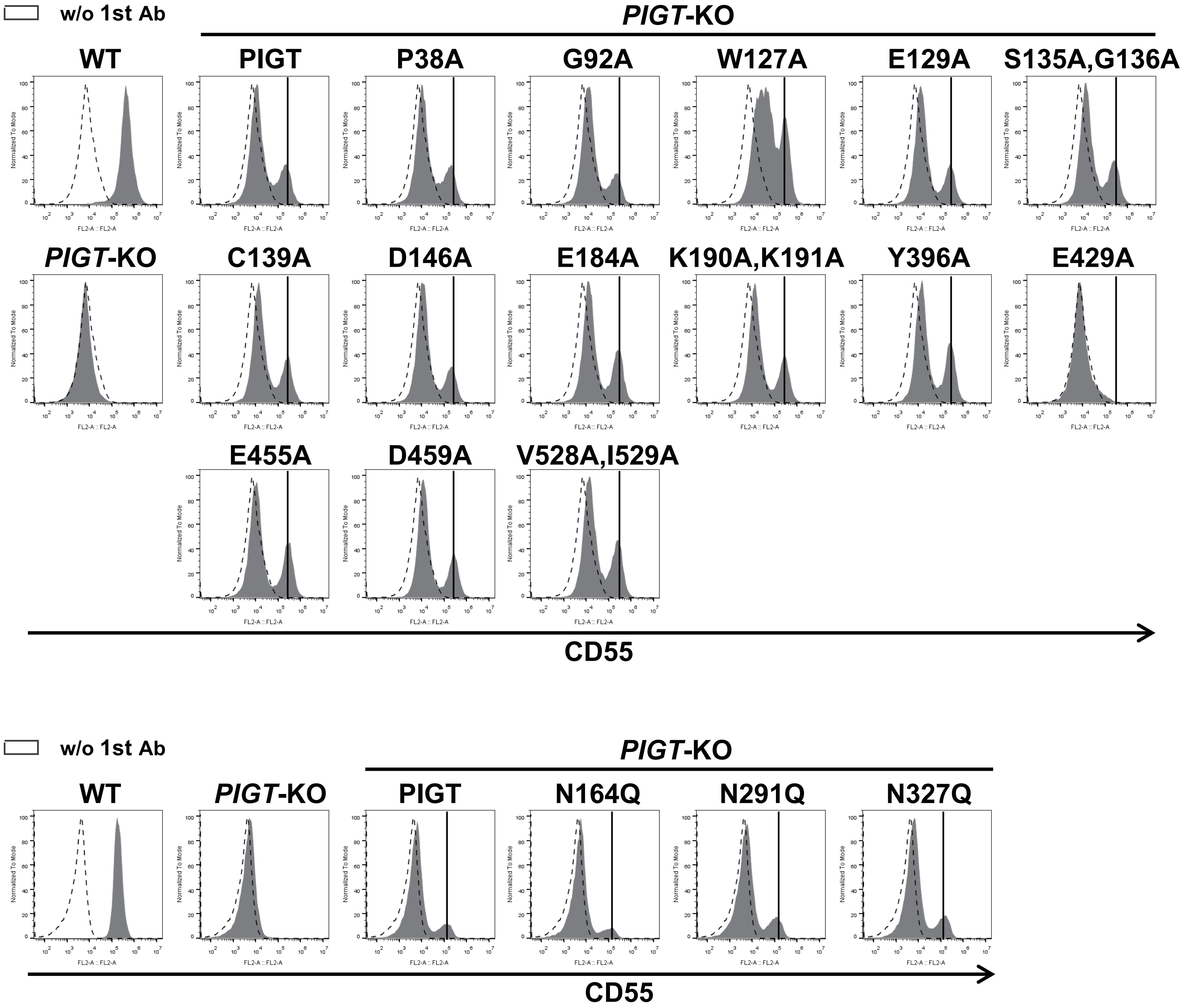

Figure S4. The effect of mutant PIGT on GPI-TA activity.

A) Sequence alignment of PIGT proteins. PIGT sequences among *H. sapiens* (UniProtKB: Q969N2), *M. musculus* (UniProtKB: Q8BXQ2), *X. tropicalis* (UniProtKB: A8WGV7), *D. rerio* (UniProtKB: E9QH65), *Bactrocera dorsalis* (UniProtKB: A0A034WF00), and *S. cerevisiae* (UniProtKB: P38875) are aligned. Conserved amino acids are colored in blue. Red stars indicate amino acids are mutated in this study, but are not affected in the GPI-TA function. The amino acids, whose mutations reduced the GPI-TA activity, are shown as green stars.

B) The indicated mutant PIGT constructs were transiently expressed in *PIGT*-KO cells. HEK293 (WT), *PIGT*-KO cells, and *PIGT*-KO cells transiently transfected with wild-type PIGT were used as controls. Surface expression of CD55 was detected using flow cytometry. In addition to the mutants in the conserved residues of PIGT, mutations in the N-glycosylation sites were detected.

Supplementary Figure S5

A

```
sp|Q96S52|PIGS_HUMAN/1-555      1 MAAAGAAATHL------EVARGKRAALFFAAVAIVLGLPLWWKTTETYRASLPYSQISGLNALQ---LRLMVPVTVVFTR---  71
sp|Q6PD26|PIGS_MOUSE/1-555      1 MAAAGAAATDL------EVVRGKRSALFFAAVAILLGLPLWWKTTETYRAPLPYSDISGLNALL---LRLMVPVTVVFTR---  71
tr|F7C443|F7C443_XENTR/1-564    1 MAAPVRSFDAI------EASRGKKVAISIALITAVLGLPLWWKTTETYRASLPYSDIRGLETLQ---LHLTVPIEIIFGK---  71
tr|F1QND8|F1QND8_DANRE/1-566    1 MLLKIVLLGIT------EKERGRYAALAIAAVVIIVGIPLWWKTTETYRAWLPFSDINELDSLQ---LRLSVDIEVVFSR---  71
tr|Q9VC10|Q9VC10_DROME/1-536    1 METSSSSPVKPRRKDKDEDKFRIGATLAFIVVIIGIGVPMWWRTTTVYRVNLPSTEILSLSKIP---IKTAVQVAIYTQQTSR  80
sp|Q04080|GPI17_YEAST/1-534     1 MSNAN---------------LRKWVGFCFVAIYLFLGVPLWYKLTTVYRASLPINYIESLQNNKFQDIHLVIPVYVKSDT---  65

sp|Q96S52|PIGS_HUMAN/1-555     72 ESVP-LDDQEKLPFTVVH-----EREIPLKYKMKIKCRFQK---------------AYRRALDHEEEALSSGSVQEAEAMLDE 133
sp|Q6PD26|PIGS_MOUSE/1-555     72 DSVP-LDDQEKLPFTVVH-----EREIPLKYKMKIKCRFQK---------------AYRRALEHEEEALSLGSVHEAEAMLAE 133
tr|F7C443|F7C443_XENTR/1-564   72 ATLT-AEQQRNVPSTGLK-----ETETVLNAQTSITRHYEF---------------KYRRTSVDEEEALGLKSLQDANNALQK 133
tr|F1QND8|F1QND8_DANRE/1-566   72 GTLT-PEQQKKIPLSHVN-----EKEHQVDAKTSLRYQYET---------------RYRTATVMEEDALNQPTAAEADLSLHT 133
tr|Q9VC10|Q9VC10_DROME/1-536   81 GQLLIAELQNAFSDNEI-------------------WSVEF---------------KQLSPTPMTQEAHTPAAL---EKLLLE 126
sp|Q04080|GPI17_YEAST/1-534    66 YRFP--DVHDAIQVQVNHLLNSQEQRVPW--------SLQVLPYNETIEQMESEGNQFHVVTLKLDEFIGYSSAYDTKE---- 134

sp|Q96S52|PIGS_HUMAN/1-555    134 PQEQAEGSLTVYVISEHSSLLPQDMMSYIGPKRTAVVRGIM------HREAFNIIGRRIVQVAQAMSLTEDVLAAALADHLPE 210
sp|Q6PD26|PIGS_MOUSE/1-555    134 PEKQAEGSLTVYVISEHSSLLPQDMMSYIGPERTAVVRGLI------HREAFNIIGRRIVQVAQAMSLTEDVLAAALADHLPE 210
tr|F7C443|F7C443_XENTR/1-564  134 LGETAHGSITIFILPESSVLLPKGIRVYIGKHRIALVRCALS-TSLGSEDIANTVYKKVEHVIQTMSFTDEAVTAALSDRIPI 215
tr|F1QND8|F1QND8_DANRE/1-566  134 LTESSCGSVVVYVIPESSVLLPEDVKVYVGQRRTALLRSPPIKSSWSLNEVISNLNPRVEQIASTMSFSHQDITAALSDRVRM 216
tr|Q9VC10|Q9VC10_DROME/1-536  127 NHVQ---SVGDFMFIEWPKLQEE---LLLTTERSALMRSDTP-----SNKIAQLLHAKILQTYR--------IN--------- 181
sp|Q04080|GPI17_YEAST/1-534   135 ----------TLVYYDDAAVLSNDLPFFVAQ--------------------------------------------TLVEHTFQ 163

sp|Q96S52|PIGS_HUMAN/1-555    211 DKWS----------AEKRRPL---KSSLGYEITFSLLNPDPKSHDVYWDIEGAVRRYVQPFLNALGAAGNFSVDSQILYYAML 280
sp|Q6PD26|PIGS_MOUSE/1-555    211 DKWS----------SDKRRPL---KSSLGYEITFSLLNPDPKSHDVHWDIEGAVQRFVQPFLNRLSVAGNFSVDSQILYYAML 280
tr|F7C443|F7C443_XENTR/1-564  216 GKFSKE------DFENSRRAF---KSSAGYEITFSLLNPDPKSHDLNWNISSASDQYIQPFLDKLQNVANFSMDSQTLYYAML 289
tr|F1QND8|F1QND8_DANRE/1-566  217 TKVTKE------SMADSMRAI---KSSPGYEITFSLLNPDPKSHSLHWDIEGALHSYIQPLLNKLAPIANFSVDSQILYYAVL 290
tr|Q9VC10|Q9VC10_DROME/1-536  182 QILS----------TDERMGAKSEAPQPAYDVIVSVLNPKPRLTHAKWNIAMAVKTYIEPWLAKVSGVSNYTVRSQWKYRVAI 254
sp|Q04080|GPI17_YEAST/1-534   164 LEWTHLNKTCEGVSTNNDVAI---SYDPNIHLSVTLLSGD--GNPVAWEIEPTLTDYFSPFRKFLSPLVNFTVDSSIVYHNDL 241

sp|Q96S52|PIGS_HUMAN/1-555    281 GVNPRF--D--SASSSYYLDMHSLPHVINPVESRLGSSA-ASLYPVLNFLLYVPELAHSPLYIQDKDGAPV-------ATNAF 351
sp|Q6PD26|PIGS_MOUSE/1-555    281 GVNPRF--D--PASSSYSLAMHSLPHVINPVESRLGSSA-ASLYPVLHFLLYVPELAHSPLYIQDKDGAPV-------ATNAF 351
tr|F7C443|F7C443_XENTR/1-564  290 GVTPRF--E--KSTSSYILNLHNLPHVINPVEARLGSSA-ASLYPVLNFLLYVPEYSHSPLYIQDKDGSRI-------PTNAF 360
tr|F1QND8|F1QND8_DANRE/1-566  291 GVNPRF--D--NSVSAYTLNADSLSHVINPVEARLGSNA-ASSNPVLNFLLYVPDAHHSPLFIRDHSRREV-------PTNAF 361
tr|Q9VC10|Q9VC10_DROME/1-536  255 EADLKQVRDQSKLGRHYALQETALPHLLTSIAQNLSAS--TTDKPAINLVVYIPPCHIAPLHIYNSKDQRL----TRNDVDAF 331
sp|Q04080|GPI17_YEAST/1-534   242 NLHSLN--G--SCT---SVTWFDLSHTIDLSE--LSSMAYYPEDSALNLAIVFPSASSSPDGLAFINGTRISDEITTLDWNSY 315

sp|Q96S52|PIGS_HUMAN/1-555    352 HSPRWGGIMVYNVDSKTYNA---SVLPVRVEVDMVRVMEVFLAQLRLLFGIAQPQLPPKCLLSGPTSEGLM--TWELDRLLWA 429
sp|Q6PD26|PIGS_MOUSE/1-555    352 HSPRWGGIMVYNVDPKIYNA---SELPVRVEVDMVRVMEVFLAQLRLLFGIAQPQVPPKCLLSGPKSEGLM--TWELDRLLWA 429
tr|F7C443|F7C443_XENTR/1-564  361 HSPRWGGIMIYNVDYGNLGE---LQFPVHIDIDMVRVMEVFLTQLRLLLGITKAAVPEDYQLESPGNEGLT--DWELDSLLWS 438
tr|F1QND8|F1QND8_DANRE/1-566  362 HSPRWGGIMVYNVNDLYGPE---SERPVDISINMAKVMGVFLAQLRLMLGVHQTHPPSGFVLQSPGSAGLA--DWELDRLMWS 439
tr|Q9VC10|Q9VC10_DROME/1-536  332 ISPPWGGFIIANPPEHVCLAAMSDQEAVPYHVSTTDNMQVMLDQLHKLLDISSELQMEGVKVVDIEQLEPR--RWEYEAYLRR 412
sp|Q04080|GPI17_YEAST/1-534   316 LVPQWGVIIINKMPLKP-NS---VISEDYL----EPMMYRFATDIFQLLGLTEGS------------QDLLSPYITIDSFKRL 378

sp|Q96S52|PIGS_HUMAN/1-555    430 RSVENLATATTTLTSLAQLLGKISNIVIKDDVASEVYKAVAAVQKSAEELA----SGH--LASAFVASQEAVTSSELAFFDPS 506
sp|Q6PD26|PIGS_MOUSE/1-555    430 RSVENLATATTTLTSLAQLLGKISNIVIKDDVASEVYRAVAAVQKAAKALA----LGH--LSSAFAASQEAVTSSERAFFDPS 506
tr|F7C443|F7C443_XENTR/1-564  439 RVVENIATVTTTLTSLAQLLDQIGNIVINDIVASEVYHAVSSVQTALSELE----AGR--LESAFQASKEAISSSEKAFFDPS 515
tr|F1QND8|F1QND8_DANRE/1-566  440 RSVENIATASTTITSLAQLLDQIGNIVINDNIALQVSSAVTSLQAAVAELD----AGN--LVFALQYSREAILASERAFFDPS 516
tr|Q9VC10|Q9VC10_DROME/1-536  413 SAIRHISTASSTLQSLIKLLDQISYIVIDDEVGAAITNSYADILAAKAALL----EHR--LADASVLAKRAFVASERGFFDAS 489
sp|Q04080|GPI17_YEAST/1-534   379 TILQNLDKATETLWSLVKLTQQFQGMSIPREVSDNVIEALDLRLQIIDLLNDPGKGGDIVWNNALHLSNELVKLCEKAFFNGE 461

sp|Q96S52|PIGS_HUMAN/1-555    507 LLHLLYFPDDQKFAIYIPLFLPMAVPILLSLVKIFLETRKSW-RKPE-------------------KTD-----          555
sp|Q6PD26|PIGS_MOUSE/1-555    507 LLHLLYFPDDQKFAIYIPLFLPMAVPILLSLVKIFQETRKSW-KKPE-------------------KID-----          555
tr|F7C443|F7C443_XENTR/1-564  516 LLHLLYFPDDQKFAIYIPLFLPMAVPIFLSLIKIAKEYKQSK-KEPL-------------------KTE-----          564
tr|F1QND8|F1QND8_DANRE/1-566  517 LLHLLYFPDDQKFAIYIPLFLPMCVPIVLSLLKIVSEVKKRRAEKQA-------------------KSD-----          566
tr|Q9VC10|Q9VC10_DROME/1-536  490 LLAQLYFPDEQKYAIYIPLFLPIMVPVLSSFNMLRGVLQARR-KEKQ---------------------S-----          536
sp|Q04080|GPI17_YEAST/1-534   462 MVQQNFFPQEHMIAVYLPLLGPISAVMFFGFYNVMKEKNQKS-KKNGTEREVAKEKLELKEAQKLHAIDGEDEL          534
```

Supplementary Figure S5

B

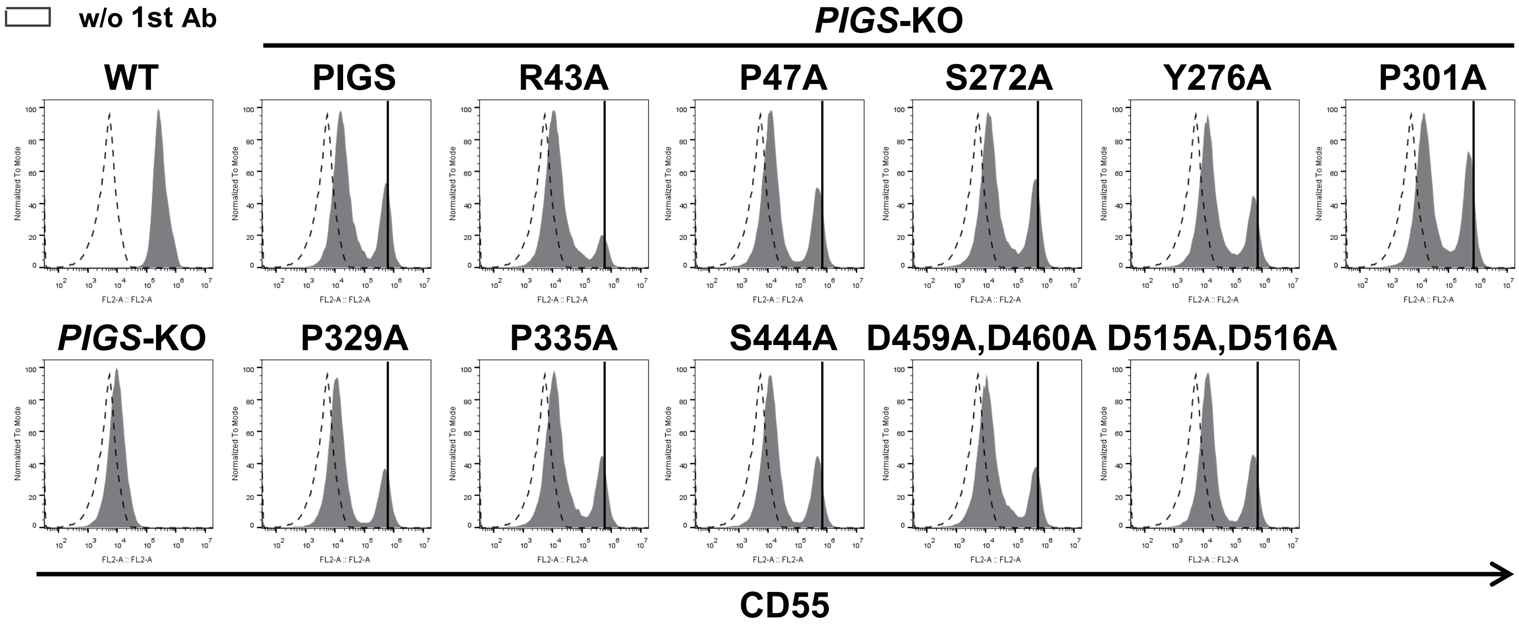

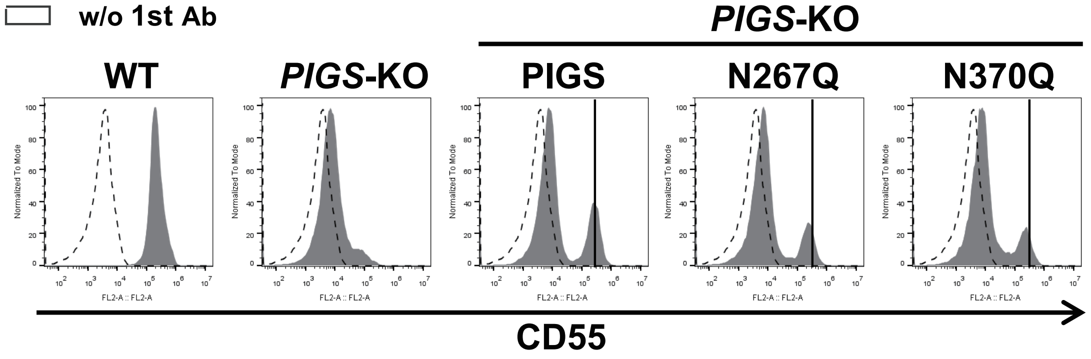

Figure S5. The effect of mutant PIGS on GPI-TA activity.

A) Sequence alignment of PIGS proteins. PIGS sequences among PIGS sequences among *H. sapiens* (UniProtKB: Q96S52), *M. musculus* (UniProtKB: Q6PD26), *X. tropicalis* (UniProtKB: F7C443), *D. rerio* (UniProtKB: F1QND8), *D. melanogaster* (UniProtKB: Q9VC10), and *S. cerevisiae* (UniProtKB: Q04080) are aligned. Conserved amino acids are colored in blue. Red stars indicate amino acids are mutated in this study, but are not affected in the GPI-TA function.

B) The indicated mutant PIGS constructs were transiently expressed in *PIGS*-KO cells. HEK293 (WT), *PIGS*-KO cells, and *PIGS*-KO cells transiently transfected with wild-type PIGS were used as controls. Surface expression of CD55 was detected using flow cytometry. In addition to the mutants in the conserved residues of PIGS, mutations in the N-glycosylation sites were detected.

Supplementary Figure S6

A

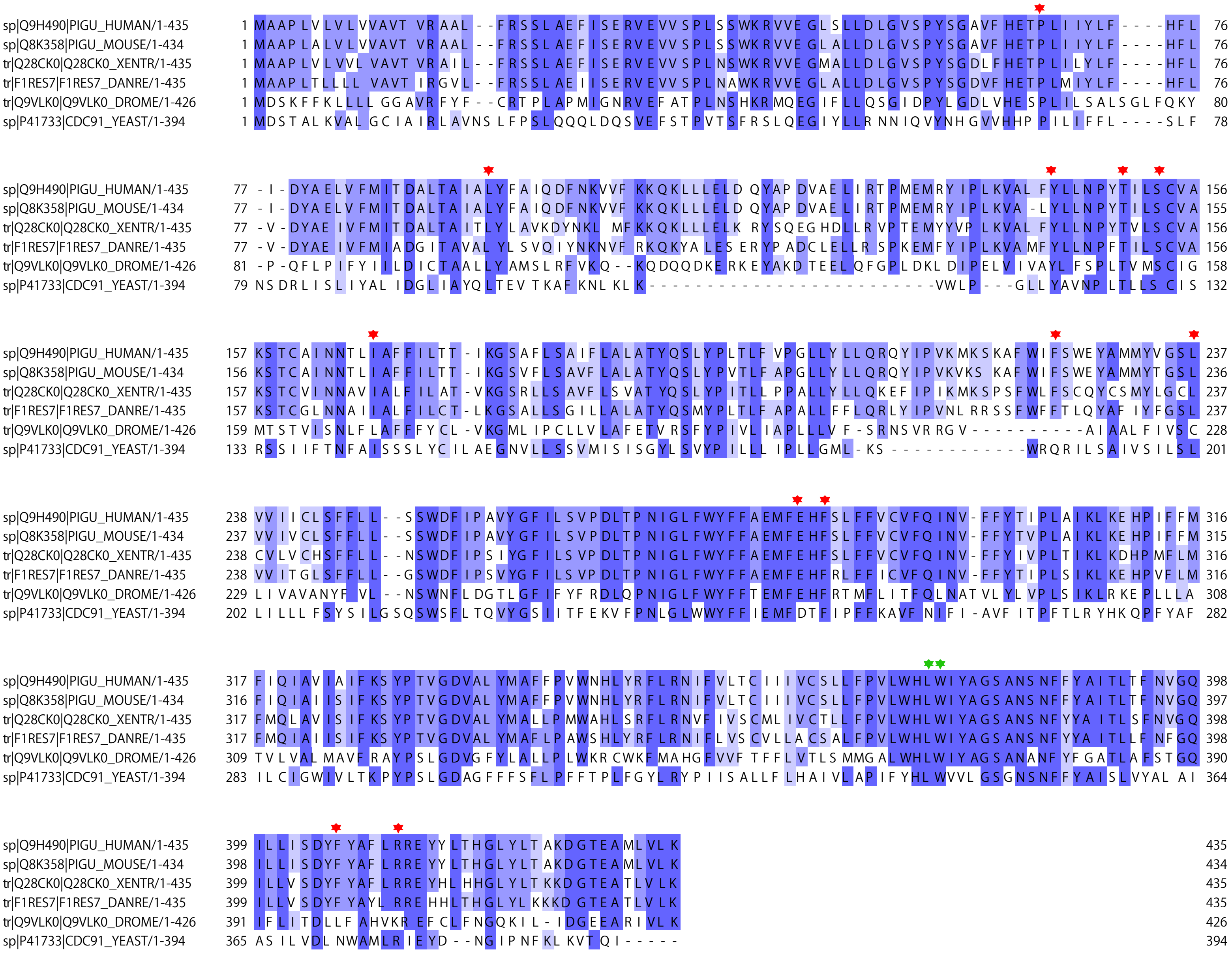

Supplementary Figure S6

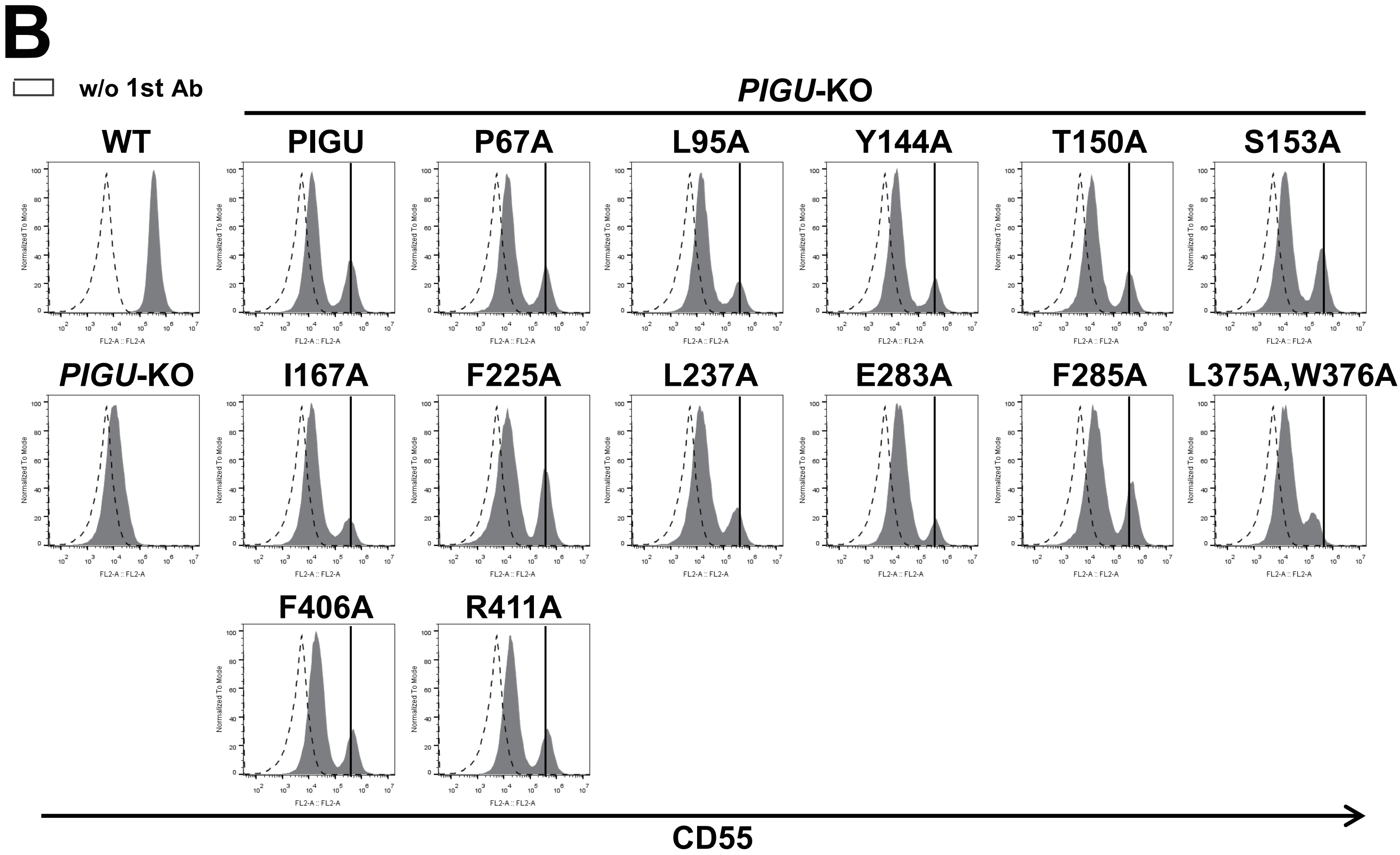

Figure S6. The effect of mutant PIGU on GPI-TA activity.

A) Sequence alignment of PIGU proteins. PIGU sequences among *H. sapiens* (UniProtKB: Q9H490), *M. musculus* (UniProtKB: Q8K358), *X. tropicalis* (UniProtKB: Q28CK0), *D. rerio* (UniProtKB: F1RES7), *D. melanogaster* (UniProtKB: Q9VLK0), and *S. cerevisiae* (UniProtKB: P41733) are aligned. Conserved amino acids are colored in blue. Red stars indicate amino acids are mutated in this study, but are not affected in the GPI-TA function. The amino acids, whose mutations reduced the GPI-TA activity, are shown as green stars.

B) The indicated mutant PIGU constructs were transiently expressed in *PIGU*-KO cells. HEK293 (WT), *PIGU*-KO cells, and *PIGU*-KO cells transiently transfected with wild-type PIGU were used as controls. Surface expression of CD55 was detected using flow cytometry.

Supplementary Figure S7

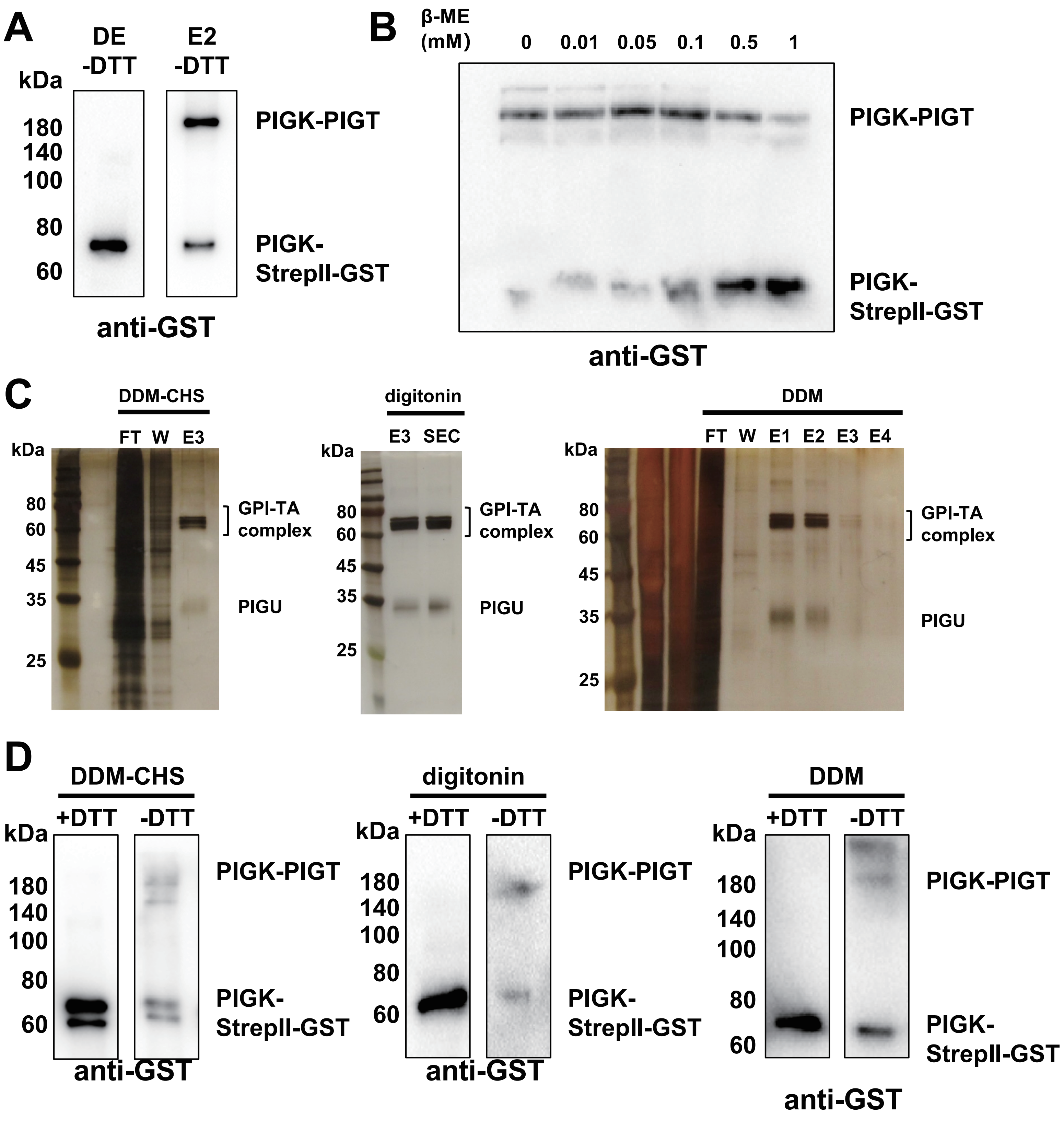

Supplementary Figure S7

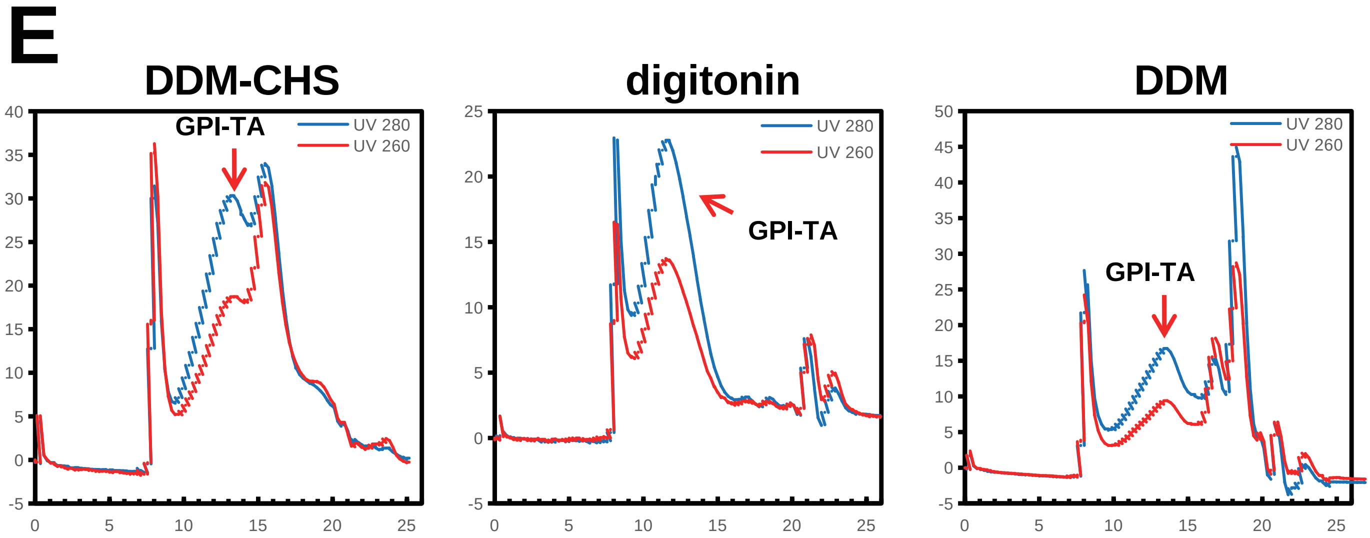

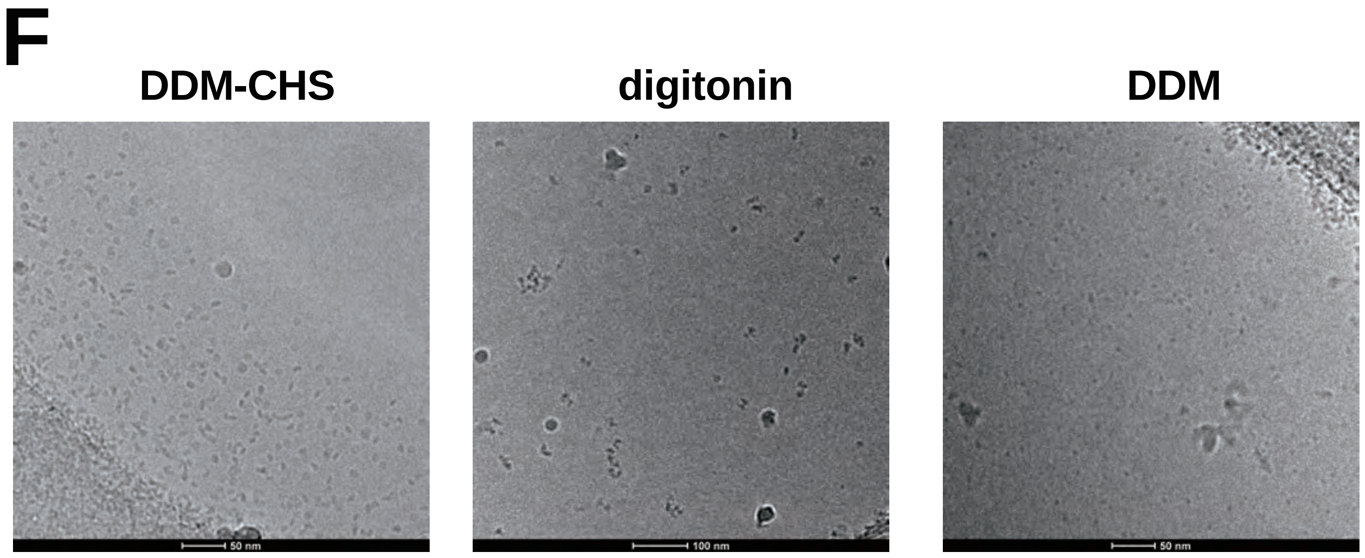

Figure S7. Optimization of large-scale purification for the GPI-TA complex.

A) PIGK-StrepII-GST containing the GPI-TA complex was purified using StrepTactin Sepharose followed by glutathione Sepharose beads (DE) or using StrepTactin Sepharose beads only (E2). PIGK linked with PIGT through a disulfide bond was disappeared when eluted with reduced glutathione from glutathione Sepharose.

B) During sample preparation, the indicated concentration of β-mercaptoethanol (β-ME) was added to analysis the effects on the intermolecular disulfide bond between PIGK and PIGT.

C) Silver staining analysis of purified GPI-TA complex by buffer containing DDM-CHS, digitonin, or DDM. All five subunits of the GPI-TA complex were detected in any buffer conditions. FT, flow through; W, wash; E1, 2, 3, and 4, elution fraction number; SEC, samples after application to size exclusion chromatography.

D) Detergent screening for GPI-TA complex purification. Using DDM-CHS, digitonin or DDM, GPI-TA containing PIGK-StrepII-GST was purified. Samples were detected under reducing (+DTT) or nonreducing (-DTT) conditions.

E) Size exclusion chromatography plots of the GPI-TA complex purified in different types of detergents.

F) EM images of the GPI-TA complex sample purified in different types of detergents, which was obtained using 200 kV cryo-EM equipped with a CCD camera.

Table S1

Table S1: LC-MS analysis of the purified GPI-TA complex.

PIGK	PIGS	PIGT	GAA1	PIGU
K.LQQDSEIM*ESSYK.E	K.AVAAVQK.S	K.ALGQLISK.Y	K.ALEGM*FR.K	K.DGTEAM*LVLK.-
K.SLCVSTPGHR.T	K.DDVASEVYK.A	K.VSIQFER.A	K.YDLVAVGK.A	K.DGTEAMLVLK.-
K.VEITTETIK.L	K.DGAPVATNAFHSPR.W	R.EGVSHYR.L	M.GLLSDPVR.R	R.TPMEM*R.Y
R.IPPSTPR.S	K.IFLETR.K ! K.LFLETR.K	R.NLNIQLK.W	R.DFAAHR.K	
R.KVEITTETIK.L	K.ISNIVIK.D !	R.TFHIEEPR.T	R.GINSFR.Q	
R.LLSDDR.S	R.LSNIVLK.E	R.TLTEPCPLASESR.V	R.VEALTLR.G	
R.TDLFQR.D	M.LGVNPR.F	R.YAVLPR.E	R.VVSTQAPDR.G	
	R.VEVDM*VR.V	R.YVSGYGLQK.G		

Table S2

Table S2: List of primers used in this study.

Primer name	Sequence (5′–3′)
PIGK-F	AAAAGCTGCGGAATTCGCCACCATGGCCGTCACCGACAGCCT
PIGK-R	CACCTCCAGCTGCAGAAAAATGAACTTCATATGCTTAATTCC
Strep-tag-F	CTGCAGCTGGAGGTGCTGTTCCAGGGCCCCAGCGCTTGGAGCCACCCCCAGTTCGAGAAAGGCGGAGGTTCCGGAGG
Strep-tag-R	ACTAGTCTAGCGGCCGCTTAGTCGACTTTTTCGAACTGAGGGTGGCTCCAAGCGCTGCCTCCAGATCCGCCTCCGGAAC CTCCGCCTTT
GST-F	GTTCGAAAAAGTCGACGGCGGAGGTGGGAGCCCTATCCTAGGTTATTGGAA
GST-R	ACTAGTCTAGCGGCCGCTTATTTTGGAGGATGGTCACCGC
K-R54A-F	TACATCCGCATTCTGGTTTAATTATCGACATGTTGC
K-R54A-R	ACCAGAATGCGGATGTACACACCAGAACAGCCC
K-N58A-F	CTGGTTTGCATATCGACATGTTGCAAATACCCTT
K-N58A-R	GTCGATATGCAAACCAGAATCGGGATGTACACA
K-R60A-F	GCACATGTTGCAAATACCCTTTCTGTTTATAG
K-R60A-R	GTATTTGCAACATGTGCATAATTAAACCAGAATCGGGATGTACA
K-H61A-F	CGAGCAGTTGCAAATACCCTTTCTGTTTATAGA
K-H61A-R	GTATTTGCAACTGCTCGATAATTAAACCAGAATCGGG
K-R74A-F	TGTCAAGGCACTAGGTATTCCTGACAGTCACATTGTC
K-R74A-R	TACCTAGTGCCTTGACACTTCTATAAACAGAAAGGGTAT
K-D79A-F	GTATTCCTGCAAGTCACATTGTCCTAATGCTTGCA
K-D79A-R	GTGACTTGCAGGAATACCTAGCCTCTTGACACTTC
K-C92S-F	ATGGCCAGTAATCCTAGAAATCCCAAACCAGC
K-C92S-R	CTAGGATTACTGGCCATATCATCTGCAAGCAT
K-E118A-F	TGATGTGGCAGTGGATTATAGAAGTTACGAGGTAACTGT
K-E118A-R	AATCCACTGCCACATCATCTCCATACACATTTAGTTCC
K-E125A-F	GTTACGCAGTAACTGTGGAGAATTTTTTACGGG
K-E125A-R	CACAGTTACTGCGTAACTTCTATAATCCACTTCCACATCA
K-E129A-F	GAGGTAACTGTGGCAAATTTTTTACGGGTATTAACTGGGA
K-E129A-R	TTTGCCACAGTTACCTCGTAACTTCTATAATCCA
K-Y160A-F	AATTGCAATGACAGGGCATGGTGGAAATGGTT
K-Y160A-R	GCCCTGTCATTGCAATTAGAATATTGCTTCTGTCATCAGAA
K-H164A-F	ATATGACAGGGGCAGGTGGAAATGGTTTCTTAAAATTTC
K-H164A-R	ACCTGCCCCTGTCATATAAATTAGAATATTGCTTC
K-D174A-F	ATTTCAAGCATCTGAAGAAATTACCAACATAGAACTCG
K-D174A-R	CTTCAGATGCTTGAAATTTTAAGAAACCATTTCCAC
K-E198A-F	GCTACAATGCACTACTGTTTATTATTGATACTTGCCAAGG
K-E198A-R	CAGTAGTGCATTGTAGCGTCTTTTCTGCCACA
K-D204A-F	ATTGCAACTTGCCAAGGAGCATCCATGTATGA
K-D204A-R	CCTTGGCAAGTTGCAATAATAAACAGTAGCTCATTGTAGCGTC
K-C206S-F	TGATACTAGCCAAGGAGCATCCATGTATGAAC
K-C206S-R	CTCCTTGGCTAGTATCAATAATAAACAGTAGCTCATTGTAGC
K-E230A-F	AGTGGGAGCAGATTCACTCTCGCATCAACCTGA
K-E230A-R	GTGAATCTGCTCCCACTTGACTACTAGCTAGAGCC
K-D231A-F	GGGAGAAGCATCACTCTCGCATCAACCTGATC
K-D231A-R	AGAGTGATGCTTCTCCCACTTGACTACTAGCTAGAGC
K-S232A-F	AGAAGATGCACTCTCGCATCAACCTGATCCTG
K-S232A-R	GCGAGAGTGCATCTTCTCCCACTTGACTACTAGCTAGA
K-D238A-F	TCAACCTGCACCTGCAATTGGAGTCCATCTTAT
K-D238A-R	TTGCAGGTGCAGGTTGATGCGAGAGTGAATCTTC
K-H244A-F	TGGAGTCGCACTTATGGATAGATACACATTTTATGTCTTGG
K-H244A-R	CCATAAGTGCGACTCCAATTGCAGGATCAGGT
K-D247A-F	GTCCATCTTATGGCAAGATACACATTTTATGTCTTGGAATTTTT
K-D247A-R	CTTGCCATAAGATGGACTCCAATTGCAGGATC
K-D289A-F	TCGCACTGCACTTTTTCAGAGGGATCCTAAAAATG
K-D289A-R	GAAAAAGTGCAGTGCGATGTCCAGGAGTAGACA
K-D302A-F	ACTGCATTCTTTGGAAGTGTACGGAAAGTGGA
K-D302A-R	CTTCCAAAGAATGCAGTTATCAGTACATTTTTAGGATCCCTC

Table S2

Primer name	Sequence (5′–3′)
HYG-ATUS-F	AGTGTTACTTCTGCTCTAAAAGCTGCGG
HYG-ATUS-R	TTTCTCTAGACTAGTCTAGCGGCCGC
A-E52A-F	ATGTCGGCAAACGCCATGGGCTCCACCATGGT
A-E52A-R	ATGGCGTTTGCCGACATGTAAGTGCGCTGGGT
A-R137A-F	GCAGCTGCCAGCACCGAGTCGCTTGTGCTCAC
A-R137A-R	TCGGTGCTGGCAGCTGCCGGGGCCCGCAGGATGCC
A-D153A-F	TGGCTCTGCATCTACCAACAGCCAGGCTGTGG
A-D153A-R	TGGTAGATGCAGAGCCACAGGGCACGGTGAGC
A-E186H187A-F	GTAACAGCAGCAGACCTTCTGGGCACTGAGGCT
A-E186H187A-R	AAGGTCTGCTGCTGTTACCAGGAAGACGATATCTTTGG
A-D188A-F	AGAACATGCACTTCTGGGCACTGAGGCTTGGC
A-D188A-R	CCAGAAGTGCATGTTCTGTTACCAGGAAGACGATAT
A-N203Q-F	ACGATGTCCAGGTCACTGGCATGCAGTCGTCT
A-N203Q-R	AGTGACCTGGACATCGTGGTAGGCTTCAAGCC
A-E226A-F	TGGCACTGAGCAGTGATGTGGTCACCAGCCTC
A-E226A-R	ATCACTGCTCAGTGCCAGGGCCACGGCTGCCTG
A-D250A-F	CAACCTTGCACTGCTCAATCTCTTCCAGACCTTC
A-D250A-R	TGAGCAGTGCAAGGTTGGGCAGCTGCCCGTTA
A-F325A-F	AATAGCGCACGCCAGTACAAGTATGACCTGGT
A-F325A-R	TACTGGCGTGCGCTATTGATGCCACGCAGGGT
A-Y328A-F	TCCGCCAGGCAAAGTATGACCTGGTGGCAGTGG
A-Y328A-R	ATACTTTGCCTGGCGGAAGCTATTGATGCCAC
A-K329A-F	CCAGTACGCATATGACCTGGTGGCAGTGGGCA
A-K329A-R	GGTCATATGCGTACTGGCGGAAGCTATTGATGC
A-E351R352A-F	TGGCAGCACTGCACCAGTCCTTCTTCCTCTACT
A-E351R352A-R	ACTGGTGCAGTGCTGCCAGGAGGTGGTTGAGCTTGC
A-N517Q-F	CTCACCCAGTTCTCACTGGGCTTCCTGCTGGC
A-N517Q-R	AGTGAGAACTGGGTGAGGGCGATGCAGCCCAG

Table S2

Primer name	Sequence (5′–3′)
T-P38A-F	GAGGAACTTGTCATCACCGCACTGCCTTCCGGGGACGTAG
T-P38A-R	CTACGTCCCCGGAAGGCAGTGCGGTGATGACAAGTTCCTC
T-G92A-F	CACCTGTCATTCACACAAGCATTTTGGAGGACCCGATACT
T-G92A-R	AGTATCGGGTCCTCCAAAATGCTTGTGTGAATGACAGGTG
T-W127A-F	AATCTGCAAAGGAGCTCAGTAATGTCCTCTCAG
T-W127A-R	GAGCTCCTTTGCAGATTTATCCACATCAGTGACAGTGTC
T-E129A-F	TTGGAAGGCACTCAGTAATGTCCTCTCAGGGATCT
T-E129A-R	TACTGAGTGCCTTCCAAGATTTATCCACATCAGTG
T-S135G136A-F	TGTCCTCGCAGCAATCTTCTGCGCCTCTCTCAACT
T-S135G136A-R	AAGATTGCTGCGAGGACATTACTGAGCTCCTTCCA
T-C139A-F	ATCTTCGCAGCCTCTCTCAACTTCATCGACTCC
T-C139A-R	AGAGAGGCTGCGAAGATCCCTGAGAGGACATTACTG
T-D146A-F	CGCCTCTCTCAACTTCATCGCATCCACCAACACAGTCACT
T-D146A-R	AGTGACTGTGTTGGTGGATGCGATGAAGTTGAGAGAGGCG
T-N164Q-F	CAGGACACTGACCACTACTTTCTGCGCTATGC
T-N164Q-R	TAGTGGTCAGTGTCCTGGGCCAGACCCAGGGGTTT
T-C182S-F	CGCGGGAGGTGGTCTCCACCGAAAACCTCACC
T-C182S-R	GGTGAGGTTTTCGGTGGAGACCACCTCCCGCG
T-E184A-F	CTGCACCGCAAACCTCACCCCCTGGAAGAAGC
T-E184A-R	TGAGGTTTGCGGTGCAGACCACCTCCCGCGGC
T-K190K191A-F	GCAGCACTCTTGCCCTGTAGTTCCAAGGCAGGC
T-K190K191A-R	ACAGGGCAAGAGTGCTGCCCAGGGGGTGAGGTTTTCG
T-N291Q-F	CAGGACCAGGAGACATTAGAGGTGCACCCACC
T-N291Q-R	AATGTCTCCTGGTCCTGGTTGTAGGTGGTGATGT
T-N327Q-F	CATGATCCAGAACTCTCGAAACCTCAACATCCA
T-N327Q-R	GAGAGTTCTGGATCATGGCGGTGTCAAGCAAG
T-Y396A-F	TATCTGCGGCTGGCAGTGCACACCCTCACCATCACC
T-Y396A-R	ACTGCCAGCCGCAGATACCAGGGTACGGTGTC
T-E429A-F	TGCAACCCCACCTCCTGGCAATGCTGATTCAGCTGCC
T-E429A-R	GGCAGCTGAATCAGCATTGCCAGGAGGTGGGGTTGCA
T-E455A-F	AAGTGGACCGCATACACGCCAGATCCTAACCATG
T-E455A-R	GTGTATGCGGTCCACTTCAGCAGCGCCCGCTC
T-D459A-F	TACACGCCAGCACCTAACCATGGCTTCTATGTCAGC
T-D459A-R	TTAGGTGCTGGCGTGTACTCGGTCCACTTCAG
T-V528I529A-F	TACAACGCAGCATGCCTCACGTGCACTGTGGTG
T-V528I529A-R	GAGGCATGCTGCGTTGTAGGGCATGCTGAAGTCC

Table S2

Primer name	Sequence (5′–3′)
T-P38A-F	GAGGAACTTGTCATCACCGCACTGCCTTCCGGGGACGTAG
T-P38A-R	CTACGTCCCCGGAAGGCAGTGCGGTGATGACAAGTTCCTC
T-G92A-F	CACCTGTCATTCACACAAGCATTTTGGAGGACCCGATACT
T-G92A-R	AGTATCGGGTCCTCCAAAATGCTTGTGTGAATGACAGGTG
T-W127A-F	AATCTGCAAAGGAGCTCAGTAATGTCCTCTCAG
T-W127A-R	GAGCTCCTTTGCAGATTTATCCACATCAGTGACAGTGTC
T-E129A-F	TTGGAAGGCACTCAGTAATGTCCTCTCAGGGATCT
T-E129A-R	TACTGAGTGCCTTCCAAGATTTATCCACATCAGTG
T-S135G136A-F	TGTCCTCGCAGCAATCTTCTGCGCCTCTCTCAACT
T-S135G136A-R	AAGATTGCTGCGAGGACATTACTGAGCTCCTTCCA
T-C139A-F	ATCTTCGCAGCCTCTCTCAACTTCATCGACTCC
T-C139A-R	AGAGAGGCTGCGAAGATCCCTGAGAGGACATTACTG
T-D146A-F	CGCCTCTCTCAACTTCATCGCATCCACCAACACAGTCACT
T-D146A-R	AGTGACTGTGTTGGTGGATGCGATGAAGTTGAGAGAGGCG
T-N164Q-F	CAGGACACTGACCACTACTTTCTGCGCTATGC
T-N164Q-R	TAGTGGTCAGTGTCCTGGGCCAGACCCAGGGGTTT
T-C182S-F	CGCGGGAGGTGGTCTCCACCGAAAACCTCACC
T-C182S-R	GGTGAGGTTTTCGGTGGAGACCACCTCCCGCG
T-E184A-F	CTGCACCGCAAACCTCACCCCCTGGAAGAAGC
T-E184A-R	TGAGGTTTGCGGTGCAGACCACCTCCCGCGGC
T-K190K191A-F	GCAGCACTCTTGCCCTGTAGTTCCAAGGCAGGC
T-K190K191A-R	ACAGGGCAAGAGTGCTGCCCAGGGGGTGAGGTTTTCG
T-N291Q-F	CAGGACCAGGAGACATTAGAGGTGCACCCACC
T-N291Q-R	AATGTCTCCTGGTCCTGGTTGTAGGTGGTGATGT
T-N327Q-F	CATGATCCAGAACTCTCGAAACCTCAACATCCA
T-N327Q-R	GAGAGTTCTGGATCATGGCGGTGTCAAGCAAG
T-Y396A-F	TATCTGCGGCTGGCAGTGCACACCCTCACCATCACC
T-Y396A-R	ACTGCCAGCCGCAGATACCAGGGTACGGTGTC
T-E429A-F	TGCAACCCCACCTCCTGGCAATGCTGATTCAGCTGCC
T-E429A-R	GGCAGCTGAATCAGCATTGCCAGGAGGTGGGGTTGCA
T-E455A-F	AAGTGGACCGCATACACGCCAGATCCTAACCATG
T-E455A-R	GTGTATGCGGTCCACTTCAGCAGCGCCCGCTC
T-D459A-F	TACACGCCAGCACCTAACCATGGCTTCTATGTCAGC
T-D459A-R	TTAGGTGCTGGCGTGTACTCGGTCCACTTCAG
T-V528I529A-F	TACAACGCAGCATGCCTCACGTGCACTGTGGTG
T-V528I529A-R	GAGGCATGCTGCGTTGTAGGGCATGCTGAAGTCC

Table S2

Primer name	Sequence (5′–3′)
T-P38A-F	GAGGAACTTGTCATCACCGCACTGCCTTCCGGGGACGTAG
T-P38A-R	CTACGTCCCCGGAAGGCAGTGCGGTGATGACAAGTTCCTC
T-G92A-F	CACCTGTCATTCACACAAGCATTTTGGAGGACCCGATACT
T-G92A-R	AGTATCGGGTCCTCCAAAATGCTTGTGTGAATGACAGGTG
T-W127A-F	AATCTGCAAAGGAGCTCAGTAATGTCCTCTCAG
T-W127A-R	GAGCTCCTTTGCAGATTTATCCACATCAGTGACAGTGTC
T-E129A-F	TTGGAAGGCACTCAGTAATGTCCTCTCAGGGATCT
T-E129A-R	TACTGAGTGCCTTCCAAGATTTATCCACATCAGTG
T-S135G136A-F	TGTCCTCGCAGCAATCTTCTGCGCCTCTCTCAACT
T-S135G136A-R	AAGATTGCTGCGAGGACATTACTGAGCTCCTTCCA
T-C139A-F	ATCTTCGCAGCCTCTCTCAACTTCATCGACTCC
T-C139A-R	AGAGAGGCTGCGAAGATCCCTGAGAGGACATTACTG
T-D146A-F	CGCCTCTCTCAACTTCATCGCATCCACCAACACAGTCACT
T-D146A-R	AGTGACTGTGTTGGTGGATGCGATGAAGTTGAGAGAGGCG
T-N164Q-F	CAGGACACTGACCACTACTTTCTGCGCTATGC
T-N164Q-R	TAGTGGTCAGTGTCCTGGGCCAGACCCAGGGGTTT
T-C182S-F	CGCGGGAGGTGGTCTCCACCGAAAACCTCACC
T-C182S-R	GGTGAGGTTTTCGGTGGAGACCACCTCCCGCG
T-E184A-F	CTGCACCGCAAACCTCACCCCCTGGAAGAAGC
T-E184A-R	TGAGGTTTGCGGTGCAGACCACCTCCCGCGGC
T-K190K191A-F	GCAGCACTCTTGCCCTGTAGTTCCAAGGCAGGC
T-K190K191A-R	ACAGGGCAAGAGTGCTGCCCAGGGGGTGAGGTTTTCG
T-N291Q-F	CAGGACCAGGAGACATTAGAGGTGCACCCACC
T-N291Q-R	AATGTCTCCTGGTCCTGGTTGTAGGTGGTGATGT
T-N327Q-F	CATGATCCAGAACTCTCGAAACCTCAACATCCA
T-N327Q-R	GAGAGTTCTGGATCATGGCGGTGTCAAGCAAG
T-Y396A-F	TATCTGCGGCTGGCAGTGCACACCCTCACCATCACC
T-Y396A-R	ACTGCCAGCCGCAGATACCAGGGTACGGTGTC
T-E429A-F	TGCAACCCCACCTCCTGGCAATGCTGATTCAGCTGCC
T-E429A-R	GGCAGCTGAATCAGCATTGCCAGGAGGTGGGGTTGCA
T-E455A-F	AAGTGGACCGCATACACGCCAGATCCTAACCATG
T-E455A-R	GTGTATGCGGTCCACTTCAGCAGCGCCCGCTC
T-D459A-F	TACACGCCAGCACCTAACCATGGCTTCTATGTCAGC
T-D459A-R	TTAGGTGCTGGCGTGTACTCGGTCCACTTCAG
T-V528I529A-F	TACAACGCAGCATGCCTCACGTGCACTGTGGTG
T-V528I529A-R	GAGGCATGCTGCGTTGTAGGGCATGCTGAAGTCC
